# Transcript Profiling Reveals the Presence of Abiotic Stress and Developmental Stage Specific Ascorbate Oxidase Genes in Plants

**DOI:** 10.3389/fpls.2017.00198

**Published:** 2017-02-17

**Authors:** Rituraj Batth, Kapil Singh, Sumita Kumari, Ananda Mustafiz

**Affiliations:** ^1^Faculty of Life Sciences and Biotechnology, Plant Molecular Biology Laboratory, South Asian UniversityNew Delhi, India; ^2^School of Biotechnology, Sher-e-Kashmir University of Agricultural Sciences and TechnologyJammu, India

**Keywords:** abiotic stress, ascorbate oxidase, genome-wide analysis, reactive oxygen species (ROS), qRT-PCR

## Abstract

Abiotic stress and climate change is the major concern for plant growth and crop yield. Abiotic stresses lead to enhanced accumulation of reactive oxygen species (ROS) consequently resulting in cellular damage and major losses in crop yield. One of the major scavengers of ROS is ascorbate (AA) which acts as first line of defense against external oxidants. An enzyme named ascorbate oxidase (AAO) is known to oxidize AA and deleteriously affect the plant system in response to stress. Genome-wide analysis of AAO gene family has led to the identification of five, three, seven, four, and six *AAO* genes in *Oryza sativa, Arabidopsis, Glycine max, Zea mays*, and *Sorghum bicolor* genomes, respectively. Expression profiling of these genes was carried out in response to various abiotic stresses and during various stages of vegetative and reproductive development using publicly available microarray database. Expression analysis in *Oryza sativa* revealed tissue specific expression of *AAO* genes wherein few members were exclusively expressed in either root or shoot. These genes were found to be regulated by both developmental cues as well as diverse stress conditions. The qRT-PCR analysis in response to salinity and drought stress in rice shoots revealed *OsAAO2* to be the most stress responsive gene. On the other hand, *OsAAO3* and *OsAAO4* genes showed enhanced expression in roots under salinity/drought stresses. This study provides lead about important stress responsive *AAO* genes in various crop plants, which could be used to engineer climate resilient crop plants.

## Introduction

Reactive oxygen species (ROS) are unavoidable consequence of aerobic metabolism. ROS are formed as a byproduct of various metabolic pathways present in different cellular compartments in plants (Foyer and Harbinson, 1994; Foyer, 1997; Sanmartin et al., 2003; del Río et al., 2006; Blokhina and Fagerstedt, 2010). Environmental stresses such as salinity, drought, chilling, metal toxicity, and UV-B radiations can intensify generation of ROS in plants by disturbing cellular homeostasis (Shah et al., 2001; Mittler, 2002; Sharma and Dubey, 2005, 2007; Hu et al., 2008; Maheshwari and Dubey, 2009; Tanou et al., 2009; Mishra et al., 2011; Srivastava and Dubey, 2011). ROS also act as secondary messengers in variety of cellular processes including environmental stresses (Desikan et al., 2001; Neill et al., 2002; Yan et al., 2007). Whether ROS act as signaling molecules or damaging molecules depends upon fine balance between ROS production and ROS scavenging. In spite of the fact that ROS are involved in signaling, they are also known to cause cellular damage (Fridovich, 1998). Increase in production of ROS during environmental stress can cause threat to cells by causing peroxidation of lipids, damage to nucleic acids, enzyme inhibition, activation of programmed cell death (PCD) pathway and ultimately causing death of the cells (Shah et al., 2001; Mittler, 2002; Verma and Dubey, 2003; Meriga et al., 2004; Sharma and Dubey, 2005; Mishra et al., 2011; Srivastava and Dubey, 2011). As ROS show multifunctional roles, it is important for the cell to control the level of ROS tightly to avoid any oxidative injury and not to get rid of them completely.

Scavenging and detoxification of ROS is achieved by antioxidant system including various enzymatic and non-enzymatic antioxidants (Noctor and Foyer, 1998). One of the non-enzymatic antioxidants vitamin C or L-ascorbic acid or ascorbate (AA; the anion of ascorbic acid) is the most abundant antioxidant found in photosynthetic tissues and plays a key role in defense against oxidative stress (Foyer et al., 1983; Smirnoff, 2000). Over 90% of the AA is present in the cytoplasm, but a considerable amount is also exported and localized in the apoplast. It is believed that apoplastic AA acts as first line of defense against the external oxidants such as ozone, SO_2_, and NO_2_ (Plöchl et al., 2000; Barnes et al., 2002).

In the apoplast, an enzyme named AAO (a glycoprotein which belongs to the blue copper oxidase enzyme family) oxidizes AA into MDA by release of an electron (Smirnoff, 2000). Unlike ascorbate peroxidase (APX) this released electron is not utilized to reduce H_2_O_2_ (Raven, 2000), rather it is accepted by O_2_ that gets reduced to H_2_O. MDA being an unstable radical undergoes rapid disproportionation to yield dehydroascorbate (DHA) and AA. The DHA can be recycled back to AA through AA-glutathione cycle (AA-GSH cycle) and MDA radical can also be recycled back to AA by the activity of an enzyme NAD(P)-dependent MDAR (Smirnoff, 2000). Transport of DHA in exchange of AA from apoplast to symplast is thought to occur via AA-DHA antiporter (or plasma membrane AA-DHA carrier), this is done to ensure continuous flux of reducing power to the cell wall (Horemans et al., 2000). Changes in AA-DHA ratio in apoplast is regulated and plays an important role in transition of cell from division to elongation state (Córdoba and González-Reyes, 1994; Kato and Esaka, 1999).

The expression of AAO is modulated by complex transcriptional and translational controls (Esaka et al., 1992), with transcript levels induced by growth promoters, e.g., auxin, (Pignocchi et al., 2003); jasmonates, (Sanmartin, 2002), and reduced by growth suppressors, e.g., salicylic acid, (Sanmartin, 2002; Pignocchi et al., 2003). The expression of AAO is induced under the influence of light and repressed in dark and this diurnal pattern of regulation is independent of circadian rhythm in *Nicotiana tabacum* (Pignocchi et al., 2003). Expression of AAO is also high in roots and young fruits (Pignocchi et al., 2003; Sanmartin et al., 2007).

Diverse roles have been ascribed to AAO enzyme in plants. AAO has been shown to maintain AA in its oxidized form which is necessary for cells to undergo mitosis (Kerk and Feldman, 1995). However, it cannot induce proliferation in non-competent cells (Citterio et al., 1994). Moreover, it is widely believed that AAO plays a critical role in cell elongation evident by its extracellular localization and high activity in rapidly expanding tissues (Esaka et al., 1992; Mosery and Kanellis, 1994; Ohkawa et al., 1994; Kato and Esaka, 1999). Tobacco (*Nicotiana tabacum*) Bright Yellow-2 protoplasts overexpressing *AAO* cDNA of pumpkin (*Cucurbita pepo*) shows cell elongation more rapidly than the other untransformed controls (Kato and Esaka, 2000). The overexpression of AAO in tobacco (Sanmartin et al., 2003) reduces stomatal aperture, consequently reducing rates of leaf water loss upon detachment, and a higher apoplast DHA content than the wild type. Transgenic tomato plants with suppressed AAO expression showed increased accumulation of AA in fruits (Zhang et al., 2011), also increased fruit yield was seen in wild type plants, where assimilates became limiting factor due to removal of leaves (Garchery et al., 2013). Enhancing AAO expression could be a possible strategy in down regulating oxygen diffusion in root nodules containing nitrogen-fixing bacteria, as well as during symbiosis with arbuscular mycorrhizal fungi (Balestrini et al., 2012). AAO overexpression was shown to delay dark induced senescence due to increase in antioxidant enzyme activity and induction in the expression of AA recycling genes such as APX and glutathione reductase (GR) (Fotopoulos and Kanellis, 2013). T-DNA mutant and antisense suppression of *AAO* gene in tobacco also leads to delayed flowering time and shorter stem length during the vegetative growth stage (Yamamoto et al., 2005).

Although, *AAO* genes have been implicated in varied cellular responses but they have not been explored with respect to their transcriptional modulation under stress conditions. Additionally, there are only few reports regarding their orthologs and paralogs in diverse plant genera. In the present report, genome-wide analysis for *AAO* genes has been carried out in *Oryza sativa, Arabidopsis thaliana, Glycine max, Zea mays*, and *Sorghum bicolor* genomes, which indicated the presence of five *AAO* genes in *Oryza sativa*, three *AAO* genes in *Arabidopsis thaliana*, seven *AAO* genes in *Glycine max*, four *AAO* genes in *Zea mays*, and six *AAO* genes in *Sorghum bicolor* genomes. Expression profiling of these genes based on publicly available microarray data has also been carried out, which indicate *AAO* are differentially regulated in response to various abiotic stresses and developmental cues. The temporal expression pattern of *AAO* genes in root and shoot tissue of rice seedlings in absence and presence of abiotic stress with the help of qRT-PCR was analyzed to comment about their transcriptional regulation in crop plant rice. The expression analysis of *AAO* genes in developmental stages and in response to various abiotic stress helped in identification of most stress responsive *AAO* genes in the five crop plants. Since *AAO* genes have been implicated in stomatal aperture reduction, increase in antioxidant activity and regulation by jasmonates as well as salicylic acid in previous studies, directed targeting of these stress responsive genes could be harnessed in engineering climate resilient crop plants.

## Materials and Methods

### *In silico* Identification of Ascorbate Oxidase Family Members

Putative *AAO* gene members in *Oryza sativa* and *Arabidopsis thaliana* were identified using protein profiles of AAO from Pfam database^1^ using HMMER 3.0 software^2^ against genome browser database TIGR Rice 6.1^3^ and TAIR^4^. *Arabidopsis* gene identifier *At5g21100* was also utilized to verify all the putative AAO proteins by searching against the annotated proteins in the whole rice and *Arabidopsis* genomes. The protein sequence of *At5g21100* was obtained from ‘The Arabidopsis Information Resource (TAIR),’ and sequence based homology search was under taken to retrieve AAO proteins from rice (TIGR) and *Arabidopsis* (TAIR) genomes using BLASTp search tool and gene search tool.

In order to search *AAO* genes in *Glycine max, Zea mays*, and *Sorghum bicolor*, the protein sequences of known AAOs with annotation score three or above were searched in UniProtKB database^5^. Sequences of six such proteins (Q40588.1, P29162, P14133.1, P37064.1, P24792.2, and Q00624.1) were retrieved and aligned through Clustal Omega^6^ and retained for analysis in Stockholm output format. Consensus sequence from these proteins was retrieved from EMBOSS CONS^7^ by providing the aligned protein sequence file. Thereafter, the entire proteome sequence was downloaded from the respective databases of *Glycine max*^8^, *Zea mays*^9^, and *Sorghum bicolor*^10^. A BLAST compatible protein database was created on our local computer from the downloaded proteome sequences and masked to remove simple internal repeats using SAGE. Standalone BLAST+ (Camacho et al., 2009) and HMMER 3.1b2^2^ were used in Linux platform on local machine for searching putative *AAO* genes in *Glycine max, Zea mays*, and *Sorghum bicolor.* The aligned sequences of pre-known AAOs obtained from UniProtKB in Stockholm format was used as query for Psi-BLAST against the masked proteome databases, separately for each species. An *e*-value cut-off of ±1*e* – 05 was taken as search threshold; redundant entries were removed from resultant list of proteins in order to create a non-redundant dataset. To further refine the search, JACK HMMER was used for iterative searching via Hidden Markov Model where consensus protein sequence of known AAOs obtained from EMBOSS CONS was used as query against the non-redundant set of protein sequences obtained from psi-BLAST. In all three plants, bit score threshold was kept 500 for HMMER search. Subsequently, the presence of three copper oxidase domains in each of the identified putative AAOs was determined through online database Pfam^11^ and SMART^12^.

### Genomic Distribution of Ascorbate Oxidase Genes on Different Chromosomes

Chromosomal locations of *AAO* genes in *Arabidopsis thaliana* were determined using chromosomal map tool of Arabidopsis information resource (TAIR^13^), and chromosomal location of *AAO* genes in rice were determined using the *Oryza sativa* genome browser^3^ along with the Ensembl genome browser^14^, while chromosomal location of *AAO* genes in *Glycine max, Zea mays*, and *Sorghum bicolor* was determined by Ensembl genome browser^14^ and mapped on their respective chromosomes. Gene duplication and their presence on duplicated chromosomal segments were also investigated. Nucleotide sequences of putative *AAO* genes in all five plants were separately aligned by Clustal Omega and a neighbor-hood joining phylogenetic tree was made without distance corrections to determine the percent identity. *AAO* genes with sequence similarity of 90% or above were considered segmentally duplicated and the series of putative genes found near each other, without any other genes in between were considered tandem duplicated genes. The relative positions of *AAO* genes and segmental duplications are shown on their respective chromosomes. Orthologs among the putative *AAO* genes in all five plants were analyzed by online tool ORCAN^15^. Genes that fall in in same orthologous groups were determined based on orthology prediction value of more than 75%. For nomenclature of *AAO* gene family in respective species, the ascorbate oxidase genes were named “AAO” with a prefix “Os,” “At,” “Gm,” “Zm,” and “Sb” for *Oryza sativa, Arabidopsis thaliana, Glycine max, Zea mays*, and *Sorghum bicolor*, respectively, and a suffix number to indicate the different members and their order of occurrence on the respective chromosomes.

### Phylogeny and Divergence of Ascorbate Oxidase Gene Family

Sequences of AAO proteins in *Arabidopsis thaliana* and *Oryza sativa* were obtained from their respective databases (TIGR and TAIR). Sequences of previously defined AAO proteins from *Nicotiana tabacum* (Q40588.1, P29162), *Cucumis sativus* (P14133.1), *Cucurbita pepo* (P37064.1), *Cucurbita maxima* (P24792.2), and *Brassica napus* (Q00624.1) were obtained from the database UniProtKB. AAO members of *Triticum aestivum, Hordeum vulgare*, and *Brassica rapa* were deduced using the same methodology followed for *Glycine max, Zea mays*, and *Sorghum bicolor.* These sequences were aligned by Clustal Omega and subjected to phylogenetic analysis by MEGA 7 (Kumar et al., 2015). Phylogenetic tree was constructed using neighbor-joining method with Poisson substitution model and complete deletion for gaps/missing data with 1000 Bootstrap replications. Alignment data is given in Supplementary Figure S1.

### Domain Search and Conserved Motif Identification in Ascorbate Oxidase Proteins

Multiple EM for Motif Elicitation (MEME) program (Bailey et al., 2006) was used for *de novo* motif detection of AAO proteins from *Oryza sativa, Arabidopsis thaliana, Glycine max, Zea mays*, and *Sorghum bicolor* with the parameters of minimum width six and maximum width of fifty amino acids. The maximum number of motifs to be searched were kept ten. Each *de novo* detected motif was further subjected for search in Interpro database^16^ to find resemblance with known domains. Consensus sequence was also separately scanned in Interpro database to find the domains present in pre-identified AAOs.

### Expression Analysis Using Microarray Data

The microarray data for the expression of *AAO* gene family members in rice during abiotic stress conditions such as cold, drought, and salt stress was retrieved from genevestigator^17^. The dataset obtained corresponds to 7-day-old IR64 rice seedlings subjected to various abiotic stress conditions.

The microarray data for *Arabidopsis AAO* genes under various abiotic stress conditions, such as cold, oxidative, drought, salt, and osmotic stresses was taken from AtGenExpress^18^. The datasets obtained were corresponding to different time points of stress, viz., 0.5, 1, 3, 6, 12, and 24 h for root and shoot tissues.

The abiotic stress microarray data of *AAO* gene family members in *Zea mays* was retrieved form genevestigator^17^. The dataset obtained corresponds to cold and drought stress. The data was retrieved for shoot samples of different varieties of *Zea mays* such as B73, OH43, and MO17 from 14-day-old seedlings subjected to cold stress of 5°C for 16 h. Data for drought stress was obtained from B73 variety of *Zea mays* wherein 4–5 days old seedlings germinated in distilled water soaked paper rolls were transferred to paper rolls soaked in PEG8000 solution with a water potential of -0.2 and -0.8 M. Pa for a period of 6 and 24 h. Data corresponds to different anatomical parts such as whole radicle, radicle tip, radicle elongation zone, stele and cortex of stress treated seedlings. Expression of *AAO* in anatomical parts such as shoot, tassel, leaf blade, ear, foliar leaf, and caryopsis were also analyzed in drought stressed plants at various developmental stages like seedling, stem elongation, inflorescence and anthesis. *Zea mays* (B73) seedlings were mostly grown till V8 developmental stage with optimal irrigation, then not irrigated completely for “n” number of days wherein number of days represents different developmental stages, such as 11, 18, 27, and 32 days represents V12 stage, V14 stage, V16 stage, and R1 stage, respectively.

Data was also retrieved for broad developmental stages of *Oryza sativa, Arabidopsis thaliana, Zea mays, Glycine max*, and *Sorghum bicolor* from genevestigator^17^. Heatmap was generated using the log_2_ signal values for dataset pertaining to stress samples of rice and *Zea mays* while mean normalized values were used for Arabidopsis to generate heatmap using MeV software package (Eisen et al., 1998).

### Plant Material and Stress Treatment for qRT-PCR Analysis

The seedlings of IR64 rice were grown under standard growth conditions in the growth chamber at 28 ± 2°C with the photoperiod of 16 h and humidity of 70–80%. The seeds were sterilized with 1% Bavistin for 20 min and allowed to germinate in hydroponic system. The germinated seeds were then supplied with yoshida media (Yoshida et al., 1971). After 10 days, rice seedlings were exposed to salinity stress (200 mM NaCl dissolved in yoshida media) and drought stress (seedlings removed from hydroponics followed by desiccation on a tissue paper towel) for a time-period of 1 and 24 h whereas untreated seedlings were used as control.

### Real-Time PCR

Total RNA was isolated from shoot and root tissue of control and stressed rice plants using IRIS kit (Bangalore, Genei) as per the manufacturer’s protocol. RNase free DNase I (Fermentas Life Sciences, USA) enzyme was used to get rid of the genomic DNA contamination in RNA samples. First strand cDNA synthesis was carried out using Maxima first strand cDNA synthesis kit for qPCR-RT (Fermentas Life Sciences, USA). Primers for real-time PCR analysis of *AAO* genes in rice were designed using NCBI primer BLAST for a product length ranging between 70 and 120 bp. The sequences for these primers are listed in Supplementary Table S1 and their binding sites are highlighted in Supplementary Figure S2. Rice β-actin and eIF-4α (eukaryotic initiation factor-4α) genes were used as reference genes for the normalization of real-time data. The PCR mixture contained 2.5 μl first strand cDNA (10 times diluted), 5 μl of 2X SYBR green PCR master mix (Fermentas Life Sciences, USA), and 2 μM of each gene-specific primer in a final volume of 10 μl. Negative template controls (NTC) were also performed for each of the primer pair. The real-time PCRs were performed employing ViiA7^TM^ real-time PCR machine (Applied Biosystems, USA). All the PCRs were performed under the following conditions: 10 min at 95°C, and 40 cycles of 15 s at 95°C, 30 s at 60°C and melt curve with single reaction cycle with following conditions 95°C for 15 s, 60°C for 1 min and dissociation at 95°C for 15 s. Three biological replicates were analyzed for each sample. The relative expression ratio was calculated using delta Ct value method (Livak and Schmittgen, 2001).

## Results

### Ascorbate Oxidase Gene Members Constitute a Small Family

In the present study, we have employed bioinformatics tools to carry genome wide analysis of *AAO* genes present in *Oryza sativa, Arabidopsis thaliana, Glycine max, Zea mays*, and *Sorghum bicolor*. Genome wide search revealed that *AAO* genes in all the five-plant species under taken in this study constitute a multigene family. Five putative *AAO* genes in *Oryza sativa*, three in *Arabidopsis thaliana*, seven in *Glycine max*, four in *Zea mays*, and six in *Sorghum bicolor* were identified, details of which are given in **Table 1**. *AAO* genes identified in all the genera analyzed here encode for respective AAO proteins with no incidence of alternative splicing, barring *At5g21105* (*AtAAO3*) gene in Arabidopsis which has three alternative spliced forms, named *At5g21105*.1, *At5g21105*.2, and *At5g21105*.3 and *GLYMA20G12230* (*GmAAO7*) in *Glycine max* with two alternative spliced forms, named *GLYMA20G12230*.2 and *GLYMA20G12230*.3.

**Table 1:** List of putative *AAO* genes in *Oryza sativa, Arabidopsis thaliana, Glycine max, Zea mays*, and *Sorghum bicolor* along with their, splice forms, nucleotide lengths, polypeptide length, CDS coordinates, chromosome number (*bp* base pair, *aa* amino acid).

	Gene	Locus identifier	Splice forms	Nucleotide length (bp)	Polypeptide length (aa)	CDS coordinates 5′–3′	Chromosome no.
***Oryza sativa***	*OsAAO1*	LOC_Os06g37080	1	1746	582	21893247–21898264	VI
	*OsAAO2*	LOC_Os06g37150	1	1902	634	21951199–21956670	VI
	*OsAAO3*	LOC_Os07g02810	1	1638	546	1055140–1058131	VII
	*OsAAO4*	LOC_Os09g20090	1	1734	578	12035268–12029912	IX
	*OsAAO5*	LOC_Os09g32952	1	1725	575	19662808–19665268	IX
***Arabidopsis thaliana***	*AtAAO1*	At4g39830	1	2415	582	18478929–18481343	IV
	*AtAAO2*	At5g21100	1	2745	573	7168184–7170928	V
	*AtAAO3*	At5g21105	3	5155	588	7172687–7177841	V
***Glycine max***	*GmAAO1*	GLYMA05G33470	1	2113	577	38106684–38109689	V
	*GmAAO2*	GLYMA08G14730	1	1962	576	10724343–10727358	VIII
	*GmAAO3*	GLYMA13G03650	1	2182	576	3663820–3672157	XIII
	*GmAAO4*	GLYMA14G04530	1	2182	581	3114029–3120767	XIV
	*GmAAO5*	GLYMA20G12150	1	2118	575	17069647–17077629	XX
	*GmAAO6*	GLYMA20G12220	1	2112	584	17148230–17152571	XX
	*GmAAO7*	GLYMA20G12230	2	1572	523	17209500–17214632	XX
***Zea mays***	*ZmAAO1*	GRMZM2G064106	1	1997	514	102651781–102655212	VII
	*ZmAAO2*	GRMZM2G163535	1	1829	574	102761977–102774858	VII
	*ZmAAO3*	GRMZM2G386170	1	1794	593	27419887–27421680	VIII
	*ZmAAO4*	GRMZM2G141376	1	2269	580	80614913–80620082	IX
***Sorghum bicolor***	*SbAAO1*	Sb02g023140	1	1713	570	56601683–56603523	II
	*SbAAO2*	Sb02g023150	1	2090	571	56649618–56655081	II
	*SbAAO3*	Sb02g023160	1	1740	579	56657530–56661262	II
	*SbAAO4*	Sb03g001450	1	1972	587	1309944–1311915	III
	*SbAAO5*	Sb10g022430	1	2000	578	50151435–50156051	X
	*SbAAO6*	Sb10g022440	1	1917	538	50171334–50174340	X

**FIGURE 1:**
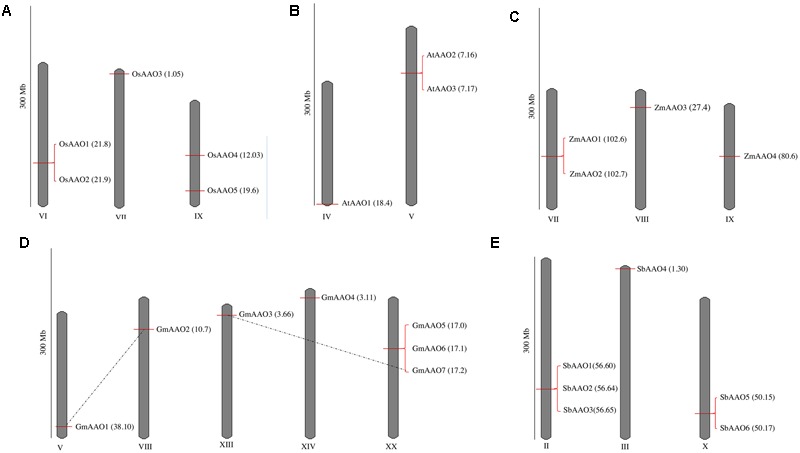
**Chromosomal distribution of *AAO* genes in diverse genera.** Chromosomal distribution of *AAO* genes in **(A)** rice, **(B)** *Arabidopsis*, **(C)** *Zea mays*, **(D)** *Glycine max*, and **(E)** *Sorghum bicolor*. Only the chromosomes having ascorbate oxidase (*AAO*) genes are shown. The scale is in (300 Mb). The chromosomal position (Mb) depicted in parenthesis along with gene name for each *AAO* is also marked as horizontal bar on the chromosome. Pairs of segmentally duplicated genes are shown by a dotted line.

Chromosomal localization analysis showed that *AAO* members were dispersed on few chromosomes, i.e., three in *Oryza sativa*, two in *Arabidopsis thaliana*, three in *Zea mays*, five in *Glycine max*, and three in *Sorghum bicolor*. A scaled representation of all *AAO* genes on their respective chromosomes has been shown in **Figure 1**. *OsAAO1* and *OsAAO2* were located on chromosome VI, *OsAAO3* was present on chromosome VII and *OsAAO4* and *OsAAO5* were present on chromosome IX (**Figure 1A**). In case of Arabidopsis, *AtAAO1* was located on chromosome IV while *AtAAO2* and *AtAAO3* were located on chromosome V (**Figure 1B**). In *Zea mays ZmAAO1, ZmAAO2* were located on chromosome VII and *ZmAAO3* and *ZmAAO4* were located on chromosome VIII and IX, respectively (**Figure 1C**). In *Glycine max GmAAO1, GmAAO2, GmAAO3, GmAAO4* were located on chromosome V, VIII, XIII, XIV, respectively, and *GmAAO5, GmAAO6*, and *GmAAO7* were located on chromosome XX (**Figure 1D**). In *Sorghum bicolor, SbAAO1, SbAAO2, SbAAO3* were located on chromosome II, *SbAAO4* was located on chromosome III and *SbAAO5, SbAAO6* were located on chromosome X, respectively (**Figure 1E**).

Few of these gene members were present in close proximity to each other on the same chromosome suggesting a possible incidence of duplication events. Analysis for segmental duplications revealed that *GmAAO1* on chromosome V could be segmentally duplicated on chromosome VIII as *GmAAO2*. *GmAAO3* on chromosome XIII could be segmentally duplicated on chromosome XX as *GmAAO7*, as these genes share more than 90% sequence identity at nucleotide level. On the other hand, *GmAAO5, GmAAO6*, and *GmAAO7* might have undergone tandem duplication as they appear as continuous cluster on the same chromosome. The sequence identity at the nucleotide level between *GmAAO5* and *GmAAO6, GmAAO6* and *GmAAO7*, and *GmAAO5* and *GmAAO7* genes is 85, 92, and 90%, respectively. Similarly, in *Sorghum bicolor SbAAO1, SbAAO2*, and *SbAAO3* genes on chromosome II and *SbAAO5* and *SbAAO6* genes on chromosome X may represent tandem duplication due to their presence in close proximity to each other. The level of sequence identity between *SbAAO1* and *SbAAO2, SbAAO2* and *SbAAO3*, and *SbAAO1* and *SbAAO3* genes is 66, 87, and 86%, respectively. *AAO* genes in *Oryza sativa, Arabidopsis thaliana*, and *Zea mays* show no incidence of segmental duplication, instead *OsAAO1* and *OsAAO2* genes on chromosome VI, *AtAAO2* and *AtAAO3* genes on chromosome V, and *ZmAAO1* and *ZmAAO2* on chromosome VII show a possible incidence of tandem duplication.

The orthologous *AAO* genes were also found among the five-plant species. *OsAAO1* gene in rice was predicted to be orthologous to *AtAAO3, GmAAO3, ZmAAO4*, and *SbAAO5*. *OsAAO4* was predicted to be orthologous to *AtAAO1, GmAAO1, ZmAAO1*, and *SbAAO2. OsAAO2* was predicted to be orthologous to *SbAAO6* while *OsAAO5* was predicted to be orthologous to *ZmAAO3* and *SbAAO*4. *ZmAAO2* in *Zea mays* was predicted to be orthologous to *SbAAO3*. No orthologs could be found for *OsAAO3* from *Oryza sativa, AtAAO2* from *Arabidopsis thaliana, SbAAO1* from *Sorghum bicolor, GmAAO2, GmAAO4, GmAAO5, GmAAO6*, and *GmAAO*7 from *Glycine max*.

### Ascorbate Oxidase Genes Are Highly Conserved across Diverse Genera

**FIGURE 2:**
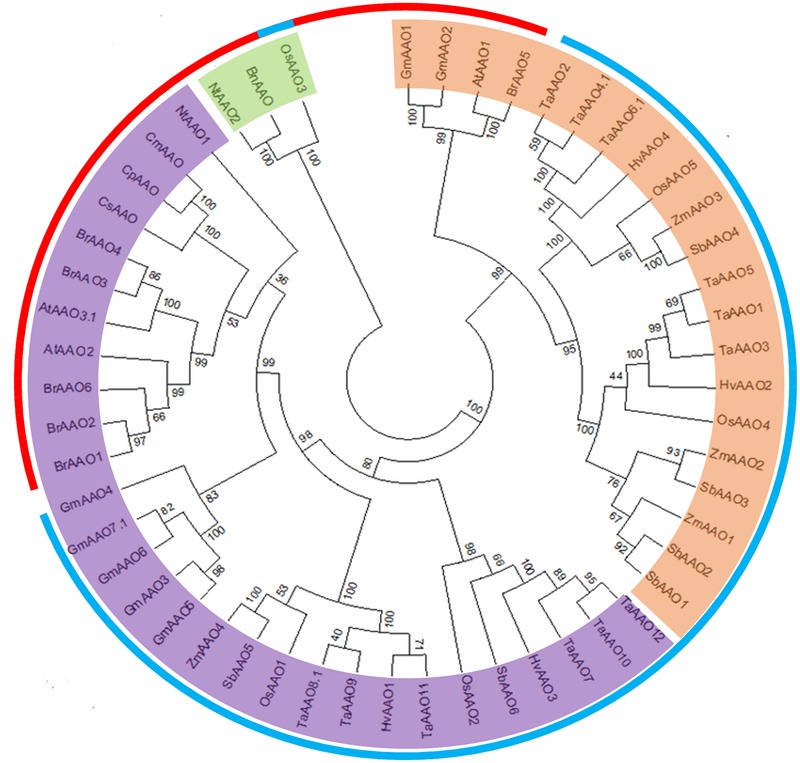
**Phylogenetic analysis of *AAO* from diverse genera.** An unrooted parsimonious tree of AAO proteins showing different clades. Clade I is marked in orange color while, clade II and clade III are marked in purple and green color, respectively. The outer circle denotes monocot (blue color) and dicot (red color) plant species taken for phylogenetic study. The tree was plotted using Mega7 software.

In order to investigate evolutionary relationship, a total of 53 AAO protein sequences from 13 different plant species were subjected to phylogenetic analysis. An unrooted tree was constructed by neighbor joining from the alignment of full-length protein sequences. The unrooted tree showed three distinct clades (**Figure 2**).

It was observed that clade I contained AAO family members mainly from monocot plants like *Oryza sativa, Zea mays, Sorghum bicolor, Triticum aestivum, and Hordeum vulgare.* However, several AAOs from these monocot species were also found in clade II and clade III. Most of AAO family members from dicot plants like *Arabidopsis thaliana, Glycine max, Brassica rapa, Nicotiana tabacum*, and *Brassica napus* were exclusively present in clade II and clade III except a small cluster of AtAAO1, GmAAO1, GmAAO2, and BrAAO5 which were the part of clade I. Although based on the signature sequence OsAAO3 has been classified as an AAO, but as per an unrooted phylogenetic tree, it was an outlier forming a third clade, clustered together with pre-known BnAAO from *Brassica napus* and NtAAO2 from *Nicotiana tabacum*, which have diverged substantially in terms of their protein sequence as compared to other AAO proteins.

All three clades contain the proteins from both dicots and monocots, suggesting that the divergence of *AAO* gene family might have occurred before the split of dicot and monocots during course of plant evolution. Additionally, it was also observed that the genes which are segmentally duplicated tend to be clustered together in the phylogenetic tree, further supporting the possibility of a duplication event.

### Identification of Conserved Motifs in Ascorbate Oxidase Proteins

**FIGURE 3:**
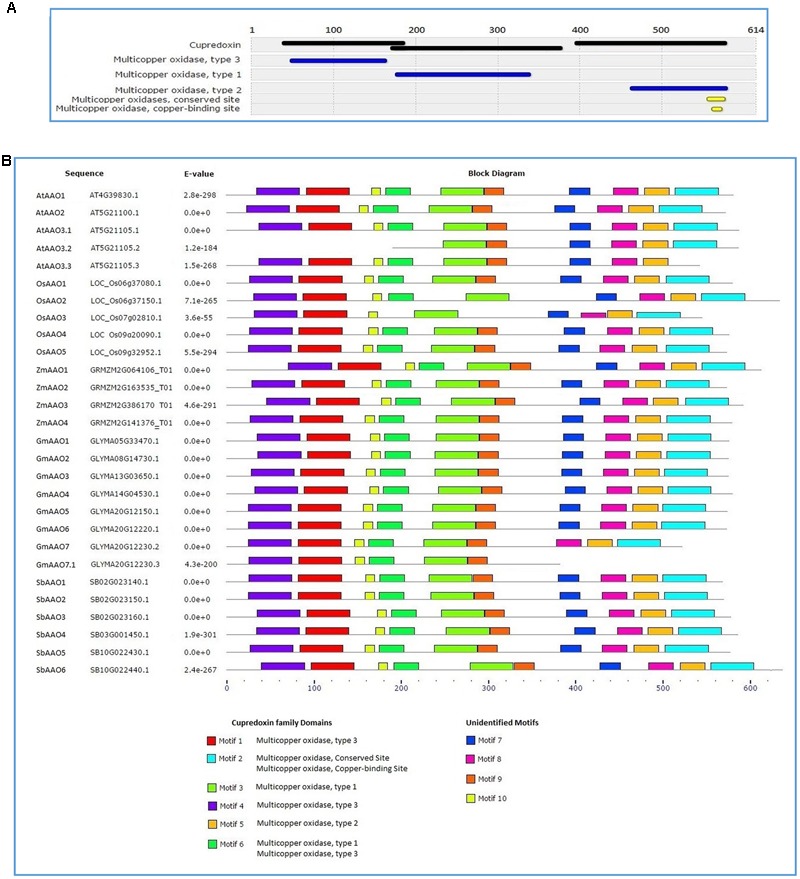
**Conserved motif identification in AAO proteins from diverse plant genera (A)** Interpro analysis revealed three types of domains as a part of cupredoxin family in previously defined AAO proteins. **(B)** *De novo* motif identification of AAO proteins; motifs 1, 2, 3, 4, 5, and 6 show resemblance to cupredoxin family and motifs 7, 8, 9, and 10 are unidentified motifs.

Scanning of consensus sequence of previously defined AAOs in Interpro identified three domains of cupredoxin family present across the entire length of its protein sequence. Each cupredoxin family domain consisted of either multicopper oxidase type 1, type 2, or type 3 domains. Multicopper oxidase conserved sites and copper binding sites were also present in sequence of multicopper oxidase type 2 domain. Pattern of domains is depicted in **Figure 3A**.

When MEME motif search tool was employed to identify the conserved motifs in AAOs identified in the present study, 10 discrete motifs were found in all putative AAOs except a spliced form of AtAAO3 and GmAAO7 (**Figure 3B**). Further, individual search of the motifs obtained from MEME in the Interpro database revealed that six motifs correspond to known domains present in copper binding AAOs while remaining four motifs did not resemble with any known domain in Interpro database. Motifs 1 and 4 were identified as the part of multicopper oxidase type 3 domain while motif 5 and motif 3 were the part of multicopper oxidase type 2 and type 1, respectively. Motif 2 displayed similarity to the multicopper oxidase conserved site and copper binding site while motif 6 resembled the sequence shared by multicopper oxidase type 1 and type 3 domains present in known AAOs (**Figures 3A,B**).

### Ascorbate Oxidase Genes Are Differentially Regulated under Stress Conditions

**FIGURE 4:**
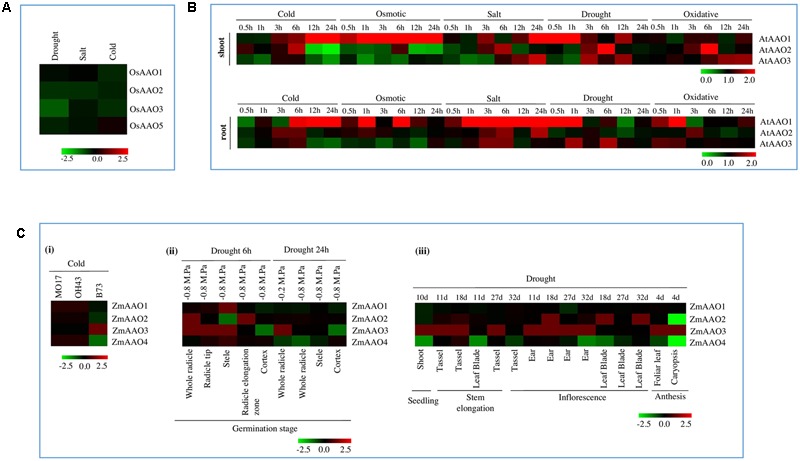
**Stress regulated expression of *AAO* genes. (A)** *Heatmap* analysis of *AAO* genes *OsAAO1, OsAAO2, OsAAO3*, and *OsAAO5* from rice in 7 days old seedlings. *Heatmap* is based on the microarray data depicting the expression profile of *AAO* genes under abiotic stress conditions such as, drought, salt, and cold **(B)** *Heatmap* analysis of *AAO* genes *AtAAO1, AtAAO2*, and *AtAAO3* from 18 days old *Arabidopsis* seedlings. Microarray data depicting expression of *AAO* gene under various abiotic stress such as osmotic, cold, salt, drought, and oxidative stress. The dataset obtained correspond to shoot and root tissue at different time point of stress such as 0.5, 1, 3, 6, 12, and 24 h with respect to control. **(C)** *Heatmap* analysis of *AAO* genes *ZmAAO1, ZmAAO2, ZmAAO3*, and *ZmAAO4* in response to abiotic stress. *Heatmap* is based on the microarray data depicting expression of *AAO* genes (i) under cold stress on different varieties of *Zea mays* (MO17, B73, and OH43), (ii) under drought stress of 6 and 24 h on different anatomical parts of germination stage with water potential ranging from -0.2 M. Pa to -0.8 M. Pa, (iii) in response to drought stress on different anatomical parts harvested from various developmental stages such as seedling stage, stem elongation, inflorescence and anthesis stage. The color bar in all figures represents the expression values, green color representing down regulation, black no change in expression and red signifies highest level of expression.

Analysis of microarray data in response to stress revealed *AAO* genes to be differentially regulated by various abiotic stresses. In rice seedlings, expression of *OsAAO1, OsAAO2*, and *OsAAO3* was downregulated in cold stress. In salt stress, expression of *OsAAO2* was downregulated while in drought stress, expression of *OsAAO2, OsAAO3*, and *OsAAO5* was downregulated (**Figure 4A**).

Microarray data analyzed for *Arabidopsis* also revealed *AAO* members to be differentially regulated at different time points in response to various abiotic stresses (**Figure 4B**). In cold stress, expression of *AtAAO1* gradually increased from early to late duration of stress in both shoot and root tissue. *AtAAO2* was briefly induced at 3 and 6 h of cold stress in shoot as well as root. Whereas, its expression was downregulated during late duration of stress in shoot and 0.5 and 12 h stress in root. *AtAAO3* expression was slightly upregulated in 3 and 6 h of stress in root and downregulated at early and late duration of cold stress in both shoot and root.

Osmotic stress lead to major upheavals in transcript abundance of *AtAAO1* gene in both root and shoot tissue. Expression of *AtAAO2* was mostly downregulated except at 6 h time point in shoot and at 1 h time point in root, where it was slightly induced. On the other hand, *AtAAO3* expression was downregulated at 3 and 12 h of stress in root and also at 1 h till 6 h of stress in root.

*AtAAO* genes were highly upregulated under salt stress in both shoot and root tissue. In shoot, expression of *AtAAO1* was high except at 0.5, 1, and 6 h of stress. Whereas, in root expression of *AtAAO1* was always high under all time-points of stress barring 0.5 h. In shoot, expression of *AtAAO2* was induced at early durations of stress and expression of *AtAAO3* was induced during late duration of stress. Whereas, in root, expression of *AtAAO2* was upregulated at both early and late duration of stress and expression of *AtAAO3* was upregulated only in early durations of salt stress.

In drought stress, expression of all *AtAAO* genes were mostly up regulated in shoot tissue, except *AtAAO2* and *AtAAO3*, which were downregulated at early duration of stress. In root tissue, expression of *AtAAO1* was mostly upregulated except at 12 h of drought stress. Expression of *AtAAO2* was mostly downregulated in root tissue, whereas, expression of *AtAAO3* was briefly induced 1 and 6 h of stress.

*AtAAO* genes mostly displayed high abundance under oxidative stress barring *AtAAO1* and *AtAAO2. AtAAO1* was downregulated at 1 and 12 h of stress in shoot and 3 h of stress in root. Whereas, *AtAAO2* was slightly downregulated during late duration of stress in both shoot and root.

All four *AAO* genes in different varieties of *Zea mays* MO17 and OH43 showed no change in expression in response to cold stress, except B73 variety, where expression of *ZmAAO4, ZmAAO2* was downregulated and *ZmAAO3* was upregulated (**Figure 4C**i). This clearly reflects upon a genotype specific regulation of *AAO* genes in *Zea mays*. In drought stress, *ZmAAO1* and *ZmAAO4* showed slight down regulation or no change in expression in different anatomical parts of various developmental stage such as germination stage, seedling stage, stem elongation, inflorescence stage and anthesis except of 6 h time point in stele where *ZmAAO1* and *ZmAAO4* showed slight up regulation (**Figures 4C**ii,iii). Expression of *ZmAAO2* was upregulated in whole radicle and radicle elongation zone stage and downregulated in stele at 6 h time point and caryopsis stage of anthesis, under drought stress. *ZmAAO3* was most stress responsive among all the four *AAOs* of *Zea mays* and its expression was mostly seen to be upregulated in cold and drought stress. Abiotic stress microarray data for *Sorghum bicolor* and *Glycine max* was not available on genevestigator, therefore it could not be included in the study.

### Ascorbate Oxidase Gene Are Differentially Regulated under Various Developmental Stages

**FIGURE 5:**
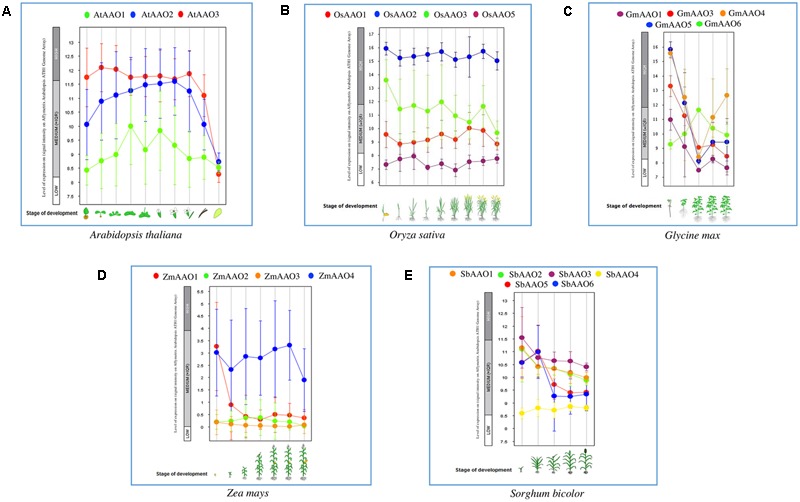
**Developmental stage specific expression of *AAO* genes.** Scatter plot displays the expression of *AAO* genes across various stages of development in **(A)** *Oryza sativa*, **(B)** *Arabidopsis thaliana*, **(C)** *Glycine max*, **(D)** *Zea mays*, and **(E)** *Sorghum bicolor*. Expression of different genes are represented by different color dots.

Since *AAO* genes are directly involved in managing ROS levels in plants, they might play a role in plant growth and development. Therefore, microarray data was also analyzed with respect to broad developmental stages of *Oryza sativa, Arabidopsis thaliana, Glycine max, Zea mays*, and *Sorghum bicolor* (**Figure 5**), to see the effect of development on transcript accumulation of *AAO* genes in plants. In *Arabidopsis, AtAAO1* expression was moderate in all developmental stages whereas expression of *AtAAO2* and *AtAAO3* was mostly moderate to high in all stages of development (such as germination, seedling, young rosette, developed rosette, bolting, young flower, developed flower and flower and silique) except for the last two stages of mature silique and senescence (**Figure 5A**).

All the rice genes showed higher relative expression at the seedling and flowering stage which are also considered as most stress sensitive stages (**Figure 5B**). *OsAAO2* and *OsAAO3* maintained a high expression in all the developmental stages while *OsAAO5* and *OsAAO1* were low expressers. Data was not available for *OsAAO4*. Vegetative and tillering stages showed relatively low abundance for all the transcripts (**Figure 5B**).

In *Glycine max* (**Figure 5C**), expression of *GmAAO3, GmAAO4*, and *GmAAO5* was high and that of *GmAAO1* and *GmAAO6* was moderate in germination stage. During shoot growth expression of *GmAAO4* and *GmAAO5* was maintained high whereas expression of *GmAAO3, GmAAO6*, and *GmAAO1* was moderate. In the flowering stage, expression of all *AAOs* dropped except for *GmAAO6* whose expression slightly increased. For fruit formation and bean development expression of all *AAOs* was low except for *GmAAO4* whose expression slightly increased.

In *Zea mays* (**Figure 5D**) expression of all *AAO* genes in germination, seedling, stem elongation, inflorescence, anthesis, fruit formation and dough stage was slightly less except for *ZmAAO1* and *ZmAAO4.* Expression of *ZmAAO1* was moderate only in germination stage, whereas expression of *ZmAAO4* was moderate in all stages of plant development. Expression of *ZmAAO4* was highest among all *AAOs*.

In germination stage of *Sorghum bicolor* (**Figure 5E**) *SbAAO3* expression was slightly high while expression of *SbAAO1, SbAAO2, SbAAO5*, and *SbAAO6* was moderate. In all other developmental stages such as stem elongation, booting, flowering and dough stage, expression of all *SbAAO* genes was moderate except for *SbAAO4*, which was a low expresser among other S*bAAOs*.

### RT-PCR and qRT-PCR Analysis Showed Shoot/Root Specific Expression of Ascorbate Oxidase Genes

**FIGURE 6:**
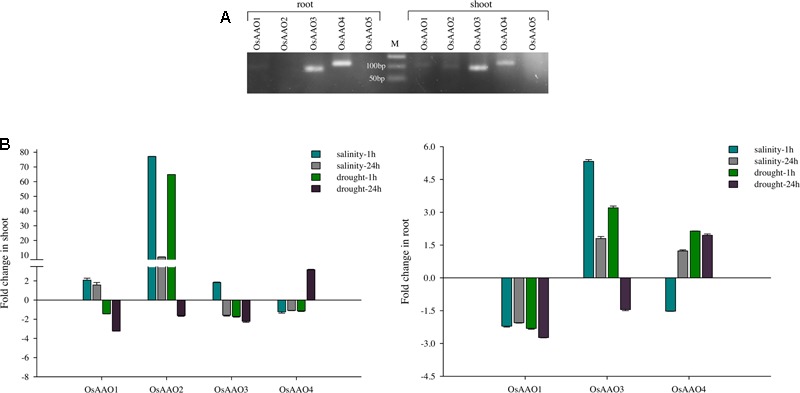
**Expression pattern of *AAO* genes in rice based on RT-PCR and qRT-PCR analysis. (A)** EtBr stained agarose gel depicting the expression of *AAO* genes in root and shoot tissue of rice seedling. Individual lanes show amplicons corresponding to *OsAAO1*–*OsAAO5* amplified from rice root and shoot tissue using gene specific real-time PCR primers. M corresponds to 50 bp ladder. **(B)** Histogram representing fold change of *OsAAO1, OsAAO2, OsAAO3*, and *OsAAO4* in 1 and 24 h stress treated shoot and root tissue of rice seedling based on qRT-PCR analysis. *OsAAO5* could not be amplified hence it was not included in real-time analysis. Real-time PCR was done with cDNA template synthesized from shoot and root tissue of 10 days old control or stressed (salinity 200 mM NaCl and drought) rice seedlings.

PCR amplification using cDNA as template was carried out to determine tissue specific (root and shoot) expression of *AAO* genes. Separate PCR reaction was set for all five *AAO* genes where pooled first strand cDNA of control and stress sample from root and shoot tissue was used as a template. It was observed that *OsAAO2* expressed specifically in shoot tissue while *OsAAO1, OsAAO3*, and *OsAAO4* genes expressed in the shoot as well as the root tissue. Unlike the other *AAO* genes, *OsAAO5* neither expressed in the shoot nor the root tissue (**Figure 6A**). However, there is a possibility that *OsAAO5* expresses in other developmental stage or tissue sample of a rice plant or the expression of *OsAAO5* might be significantly low to be detected as observed in the microarray based expression analysis wherein *OsAAO5* displayed very low expression across various developmental stages in this study.

The relative transcript level of *OsAAO1, OsAAO2, OsAAO3*, and *OsAAO4* was determined in response to salinity and drought stress in shoot and root tissue of rice seedlings. *OsAAO5* gene could not be amplified from both shoot and root cDNA used in this study; therefore, it is not included in the expression analysis. The real-time data reveals differential tissue specific stress inducibility of the members of *AAO* gene family in *Oryza sativa* IR64. *OsAAO2* was found to be specifically expressed in shoot and it was upregulated under both drought and salinity stress treatment except in tissue subjected to late durations of drought stress (24 h) where it was downregulated. Expression of *OsAAO1* was upregulated in shoot tissue in response to both early and late durations of salinity stress while it was downregulated at both time points of drought stress. *OsAAO3* and *OsAAO4* genes were upregulated only in response to early duration of salinity stress and late duration of drought stress, respectively. Both the genes were downregulated in shoots in response to all the other stress conditions analyzed in this study (**Figure 6B**). *OsAAO1* was downregulated in root tissue under both early and late duration of salinity and drought stress. *OsAAO3* gene showed enhanced expression in roots under both salinity (early and late duration) and drought (early duration) while it was downregulated during late durations of drought stress. *OsAAO4* showed enhanced expression under drought stress conditions as well as in late duration (24 h) of salinity (**Figure 6B**).

## Discussion

Availability of whole genome sequences of model and important food crops has served as a useful platform to carry out comprehensive analysis of gene families, e.g., study of glyoxalase gene family in rice, *Arabidopsis* (Mustafiz et al., 2011), soybean (Ghosh and Islam, 2016), analysis of CBS domains containing proteins (CDCPs) in *Arabidopsis* and rice, (Kushwaha et al., 2009), analysis of F-box protein, auxin-responsive SAUR gene family and homeobox genes in rice (Jain et al., 2006, 2007, 2008), analysis of *CaHsp20* gene family in pepper (Guo et al., 2015), study of *AAAP* (amino acid/auxin permease) gene family in maize (Sheng et al., 2014) and analysis of *PIN* auxin transporter gene family in soybean (Wang et al., 2015) etc. In the present study, we attempted a genome-wide analysis of *AAO* gene family in *Oryza sativa, Arabidopsis thaliana, Glycine max, Zea mays*, and *Sorghum bicolor* and as well as analysis of their transcript abundance specific to abiotic stress and developmental stages. *AAO* genes form a small gene family in plants. *AAO* multigene families identified in the five-plant species under study were found to have variable number of members, viz., five in *Oryza sativa*, three in *Arabidopsis thaliana*, seven in *Glycine max*, four in *Zea mays*, and six in *Sorghum bicolor*. Variation in gene number of *AAO* among the five-plant species could be due to variation in number of duplication events, for example, *AAO* genes in soybean show more duplication events and constitutes a larger *AAO* gene family as compared to *Arabidopsis, Zea mays*, and *Oryza sativa*. Gene family size variation is a common phenomenon in plant genera which could be attributed to gene duplication, deletion, pseudogenization, and/or functional diversification (Zhang et al., 2010). AAOs are best described from plants (Kues and Ruhl, 2011), however, much is not known about their precise biological function. Genes for putative AAOs have also been reported from some fungi (Hoegger et al., 2006). Plant AAOs have been implicated in oxygen homeostasis and ROS balancing (De Tullio et al., 2004; Semchuk et al., 2009), various stress reactions (Sanmartin et al., 2003; Caputo et al., 2010), defense (Barbehenn et al., 2008), growth and cell wall formation (Ros-Barceló et al., 2006; Díaz-Vivancos et al., 2010), and signaling (Pignocchi et al., 2003; Fotopoulos et al., 2008). Based on the previous studies, it has been established that they belong to multiple copper oxidase family proteins. To further comment upon the type of copper oxidase domain, AAO proteins were investigated for the type of copper binding domains.

Similar *de novo* identified motifs are present and conserved across all of the putative AAOs considered in this study. Some of these motifs correspond to one of the three conserved domains (multicopper oxidase types 1, 2, and 3) identified in pre-defined AAO proteins. The presence and similar pattern of the conserved domains/motifs in all putative AAOs suggests the high structural similarity among the *AAO* gene family members found in *Oryza sativa, Arabidopsis thaliana, Glycine max, Zea mays*, and *Sorghum bicolor*. Furthermore, the resemblance of *de novo* identified motifs in putative AAOs with that of known domains in pre-identified AAOs suggests that all *AAO* genes taken in this study are functionally similar and can be active AAOs. Although these gene members showed known copper binding motifs, some unreported motifs were also found in these proteins.

It has been postulated that the three-domain multi-copper blue proteins, such as AAO, have evolved by a single domain addition to the two-domain protein. Alternatively, it has been suggested that AAO could have evolved from a six-domain protein by replacing domains 3 through 5 with a short linker (Nakamura et al., 2003). These postulations are further strengthened by the presence of a small stretch of non-conserved linker like sequence between multicopper oxidase domain types 1 and 2 in pre-defined AAOs as well as between motifs 7 and 9 in all putative AAOs depicted in **Figures 3A,B**.

An important aspect of response to stress occurs at transcriptional level, which alters gene expression (Tester and Davenport, 2003). In several previous studies, AAO activity and expression are closely co related with light, salicylic acid, auxin, and jasmonates (Sanmartin, 2002). Light driven ROS production detrimentally affects the redox balance of photosynthetic tissues and also the overall plant growth and development (Foyer and Shigeoka, 2011). AA acts as one of the most important non-enzymatic, water soluble antioxidant in plants to metabolize ROS (Foyer et al., 1983; Smirnoff, 2000). However, AAO leads to oxidation of AA and therefore preventing the detoxification of ROS (Smirnoff, 2000). These findings suggest AAO to be involved in negative regulation of stress response. However, no direct correlation has been established between environmental perturbations and AAO activity or transcriptional activity. For this reason, we wanted to study if *AAO* genes are differentially regulated in response to abiotic stress, viz., salinity and drought conditions. Expression pattern of *AAO* genes retrieved from publically available microarray data and revalidated through RT-PCR as well as qRT-PCR suggested these genes to be strongly stress responsive showing a genotype/genus and tissue specific temporally regulated expression. *OsAAO2* is one of the most stress responsive gene in shoot tissue of rice while *OsAAO3* and *OsAAO4* show high expression in root tissue in response to salinity and drought stress, as analyzed by qRT-PCR. *AtAAO1* was highly upregulated in shoot and root in response to abiotic stress and *AtAAO3* displayed high expression in various developmental stages. In *Zea mays, ZmAAO3* is the most stress responsive gene as seen in both cold and drought stress. *OsAAO2, OsAAO3* and *OsAAO4* from *Oryza sativa, AtAAO1 and AtAAO3* from *Arabidopsis* and *ZmAAO3* from *Zea mays* can serve as a good candidate for raising stress tolerant transgenic crops.

**FIGURE 7:**
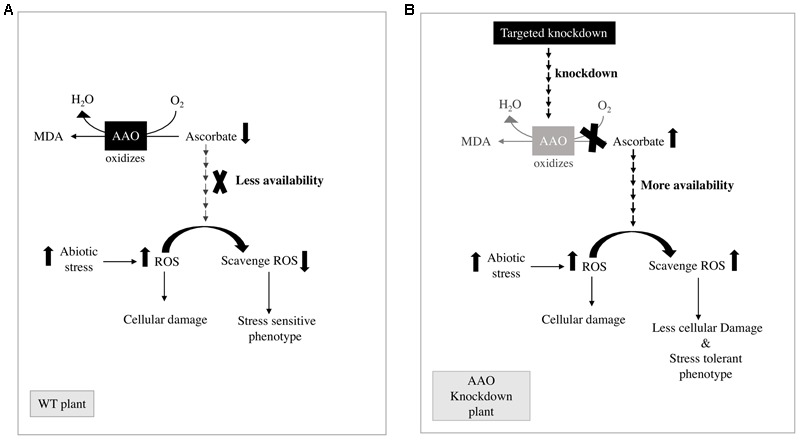
**Mechanistic details of possible pathway of AAO action in regulating stress phenotype of plants. (A)** Reduced AA availability due to the oxidizing activity of AAO leads to stress sensitive phenotype in WT plants. **(B)** Enhanced AA availability due to the knock down of *AAO* gene leads to stress tolerant phenotype in AAO knock down plants.

Based on the previous reports as depicted in **Figure 7A**, AA works in close coordination with GSH in ASA-GSH cycle (Foyer and Halliwell, 1976) and water–water cycle (Asada, 1999) to metabolize ROS. Therefore, the redox state of AA is of utmost importance to prevent ROS induced cell injury. However, the cell wall localized enzyme AAO leads to oxidation of AA, to form unstable MDHA in turn reducing the AA/DHA ratio (Smirnoff, 2000) hence affecting the detoxification of ROS leading to more damage to plants (**Figure 7A**). Additionally, overexpression of AAO in tobacco reduces capacity of plant to scavenge ROS in leaf apoplast due to oxidation of AA in this compartment consequently leading to enhanced sensitivity to ozone (Sanmartin et al., 2003). On the other hand, if the activity of AAO is suppressed, the availability of AA in its reduced form is increased. This increased level of AA in its reduced form helps scavenge more ROS as compared to wild type plants and plants show stress tolerant phenotype (**Figure 7B**). It has also been reported in tobacco and *Arabidopsis* that if the *AAO* gene expression is suppressed, it grants resistance to oxidative damage brought about by methyl viologen or H_2_O_2_ (Yamamoto et al., 2005). Furthermore, in the same study it was also shown that AA/DHA ratio was higher in case of antisense-AAO tobacco plants and *Arabidopsis* T-DNA AAO mutant plant than those of wild type plants during growth under salt stress conditions, whereas overexpressing of AAO in tobacco leads to greater damage of transgenic plants as compared to wild type plants (Yamamoto et al., 2005). In plants, such as tobacco and *Arabidopsis* it has been seen, that the level of H_2_O_2_ decreased on suppressing the activity of *AAO* gene. It was also seen in previous studies that antisense suppression of the *AAO* gene in tobacco enhanced percentage germination and increased seed yield at high salinity conditions (Yamamoto et al., 2005). These findings suggest that the targeted knock down of *OsAAO2, OsAAO3*, and *OsAAO4* in rice which have been found to show steep changes in stress responsive transcript accumulation can be a possible strategy to engineer stress tolerance in rice. It would be interesting to study the changes in redox state of AA in engineered plants and its effect on the level of ROS and damage caused by them.

## Author Contributions

The idea, concept, design of experiments and manuscript preparation are done by AM. SK has done the bioinformatics work and contributed in preparing the manuscript. RB has done the wet lab experiments and also contributed in manuscript preparation and KS has contributed in bioinformatics work.

## Conflict of Interest Statement

The authors declare that the research was conducted in the absence of any commercial or financial relationships that could be construed as a potential conflict of interest.

## Footnotes

1 http://pfam.sanger.ac.uk/

2 http://hmmer.org/

3 http://rice.plantbiology.msu.edu/

4 https://www.arabidopsis.org

5 http://www.uniprot.org/uniprot/

6 http://www.ebi.ac.uk/Tools/msa/clustalo/

7 http://www.bioinformatics.nl/cgi-bin/emboss/cons

8 ftp://ftp.ensemblgenomes.org/pub/plants/release-33/fasta/glycine_max/pep/

9 http://www.plantgdb.org/XGDB/phplib/download.php?GDB=Zm

10 ftp://ftpmips.helmholtz-muenchen.de/plants/sorghum/genes/

11 http://pfam.xfam.org/

12 http://smart.embl-heidelberg.de/

13 https://www.arabidopsis.org/jsp/ChromosomeMap/tool.jsp

14 http://plants.ensembl.org/index.html

15 http://www.combio.pl/orcan

16 https://www.ebi.ac.uk/interpro/

17 https://genevestigator.com/gv/start/start.jsp

18 http://jsp.weigelworld.org/expviz/expviz.jsp

## Abbreviations

AA: ascorbate
AAO: ascorbate oxidase
MDA: monodehydroascorbate
MDAR: monodehydroascorbate reductase
