# Supplementary material for: Transcript Profiling Reveals the Presence of Abiotic Stress and Developmental Stage Specific Ascorbate Oxidase Genes in Plants
Source: Front Plant Sci. 2017 Feb 17;8:198. doi: 10.3389/fpls.2017.00198 (PMC5314155; doi:10.3389/fpls.2017.00198)
Supplement: Supplementary file 1 [file Table_1.DOCX]

**Transcript profiling reveals the presence of abiotic stress and developmental stage specific ascorbate oxidase genes in plants**

**Rituraj Batth, Kapil Singh, Sumita Kumari ^*^, Ananda Mustafiz^*^**

^*^Correspondence: Ananda Mustafiz: [amustafiz@sau.ac.in](mailto:amustafiz@sau.ac.in)

^*^Correspondence: Sumita Kumari: [sumitaslsjnu@gmail.com](mailto:sumitaslsjnu@gmail.com)

**Table S1:** List of primers and their respective sequences for qRT-PCR of rice *AAO* genes. Here “Os” stands for *Oryza sativa* and AAO stands for ascorbate oxidase.

| Gene name | Locus identifier | Primer name | Primer sequence |
| --- | --- | --- | --- |
| *OsAAO1* | LOC_Os06g37080 | A1real-F  A1real-R | AGCTGCTCAACACGCAGAAC  ATCTTGAGGGACCCCAGGTA |
| *OsAAO2* | LOC_Os06g37150 | A2real-F  A2real-R | GGTGATGATCGGGATAAACG  CGGTGTGCATCTTGTTGTTC |
| *OsAAO3* | LOC_Os07g02810 | A3real-F  A3real-R | GCCAGCAGCTGTACATGAGA  CTCTACCGCAGAGTATGGCG |
| *OsAAO4* | LOC_Os09g20090 | A4real-F  A4real-R | CAACCTCAGGGACCCAATCA  CCTCGATGTGGCAGTGGAAC |
| *OsAAO5* | LOC_Os09g32952 | A5real-F  A5real-R | CACCAAGGGCCATCACTACT  TGGACGTTCGTCACCATGA |
