# Supplementary material for: Transcript Profiling Reveals the Presence of Abiotic Stress and Developmental Stage Specific Ascorbate Oxidase Genes in Plants
Source: Front Plant Sci. 2017 Feb 17;8:198. doi: 10.3389/fpls.2017.00198 (PMC5314155; doi:10.3389/fpls.2017.00198)
Supplement: Supplementary file 2 [file Data_Sheet_1.DOCX]

**Transcript profiling reveals the presence of abiotic stress and developmental stage specific ascorbate oxidase genes in plants**

**Rituraj Batth, Kapil Singh, Sumita Kumari ^*^, Ananda Mustafiz^*^**

^*^Correspondence: Ananda Mustafiz: [amustafiz@sau.ac.in](mailto:amustafiz@sau.ac.in)

^*^Correspondence: Sumita Kumari: [sumitaslsjnu@gmail.com](mailto:sumitaslsjnu@gmail.com)

**Figure S1:** Multiple sequence alignment of AAO protein sequence from *Oryza sativa* (OsAAO), *Arabidopsis thaliana* (AtAAO), *Glycine max* (GmAAO), *Zea mays* (ZmAAO), *Sorghum bicolor* (SbAAO), *Nicotiana tabacum* (NtAAO), *Brassica rapa* (BrAAO), *Hordeum vulgare* (HrAAO), *Brassica* *napus* (BnAAO), *Triticum aestivum* (TvAAO), *Cucurbita maxima* (CmAAO), *Cucumis sativus* (CsAAO) and *Cucurbita pepo* (CpAAO).

NtAAO2 -----------------------------------------------MGSGKVTFVA--L

BnAAO -----------------------------------------------MRGVKLLAACLYL

OsAAO3 ------------------------------------MAPP-------PAAAAALAAC--I

GmAAO1 -----------------------------------MVELR-L--SIRALQLPTLLILCFF

GmAAO2 -----------------------------------MVELGLI--SLRALQLPRLLILCFF

AtAAO3 -----------------------------------MMRP-KR--SSDTVHVFNLMVLCFI

BrAAO5 -----------------------------------MMRS-CR--FSDTSHVFTLMFICFI

HvAAO2 -----------------------------MSGGGGA----ME--SSLI-AKHLLLCCLFL

TaAAO5 ------------------------------MRGGGA----ME--SSLLGANPRLLCCLFL

TaAAO1 -----------------------------MSGGGAA----ME--SSLLCAKQLLLCCLFL

TaAAO3 ----------------------------------------ME--SSLLGAKQLLLCCLFL

OsAAO4 ----------------------------------------------MMRCSDRLLCSLFL

ZmAAO2 ----------------------------------MALLP--G-------AARRLLCSLFL

SbAAO3 ----------------------------------MALLP--R--AAPARHAQRLLCSLFL

ZmAAO1 TGITAPLESRHIAAACLCTRAQSGTPISHIIGSSSCAAPHCV--DPAMGCPPRLLCCLFL

SbAAO2 -----------------------------------------------MGRLPWLLCCLFL

SbAAO1 -----------------------------------------------MGWQPRLLCCLFL

ZmAAO3 ----------------------ARRSFSSRSGL--TMSAP-R--QPLCKLATAVHHLLLC

SbAAO4 ------------------------------------MAPP-R--RPLRMPAAVLLLLVLC

OsAAO5 ----------------------------------------------MRTWRLAVL-ACLC

HvAAO4 ------------------------------MAG--TMLPP-P--RPLLAAAGALS-FCLC

TaAAO6.1 ------------------------------------MLPS-A--RPLLAPAAA----VLC

TaAAO2 ------------------------------------MLPS-A--RPLLAA-AA----VLC

TaAAO4.1 ------------------------------------MLPS-A--RPLLAA-AA----VLC

AtAAO2.1 -----------------------------------MSYDEH---TSSSFTYISQMGVWWI

BrAAO3 ------------------------------------------------------MGVWWI

BrAAO4 ------------------------------------------------------MGMWWI

AtAAO1 ----------------------------------------------------MAVIVWWL

BrAAO6 ----------------------------------------------------MSVIVWWL

BrAAO1 ----------------------------------------------------MSVVVWWL

BrAAO2 ------------------------------------------------------------

TaAAO8.1 -----------------------------------------------MRALF-AWCALLL

HvAAO1 --------------------------------------------MRAPVAFF-AWCALLL

TaAAO9 -----------------------------------------------MRALF-AWCALLL

TaAAO11 -----------------------------------------------MRALF-AWCALLL

OsAAO1 --------------------------------------------MRLS-SLLFLVCFFTV

ZmAAO4 --------------------------------------------MRLPLPLLALVCCALM

SbAAO5 --------------------------------------------MRLPL-LGALVCCALM

CsAAO -----------------------------------MAKVADKPFFPKPFLSFLVLSIIFG

CpAAO ------------------------------------------------------------

CmAAO --------------------------------MLQMGKAREP-----NFLILFFFGLILA

NtAAO1 -------------------------------------------MASLGFLFFFLLPLILL

GmAAO4 -----------------------------------MSKS---NNNIMSFKALTLWCIL--

GmAAO7.1 ----------------------------------------------MSLKALFVWCIIW-

GmAAO6 ----------------------------------------------MSLKALFVGCIIW-

GmAAO3 -------------------------------------------MVTMGLKALFVWCIIW-

GmAAO5 ----------------------------------------------MGLKALFVWCIIW-

SbAAO6 ----------------------------------------M-GKPYVSLAVQLLICCALM

OsAAO2 ----------------------------------------------------MAAAVQLL

HvAAO3 -----------------------------------MARPHT--RSESPLVVHLLLCCTFL

TaAAO7 -----------------------------------MTRPHT--GSEPPLVMHLLLCCTFL

TaAAO10 -----------------------------------MTRPHT--GSESPLVVHLLLCCTFL

TaAAO12 -----------------------------------MTRPHT-SSSESPLMVHLLLCCTFL

NtAAO2 LLCLS--------VGVIAEDPYLYFNWNVTYGTIAPLG-VPQQGILINGQFPGPRINCTS

BnAAO AAAAT--------VVVHAEDPYFHHVWNVTYGTASPLG-VPQQVILINGQFPGPNINSTS

OsAAO3 LAVAA--------TLAGADDPYRFFTWNVTYGSINPLGSTPQQGILINGQFPGPRIDCVT

GmAAO1 LILG---------NFHKAEARIRHHKWEVKYEFRSPDC-FKKLVITINGKTPGPTIQAQE

GmAAO2 VILG---------NFHKAEARIRHYKWEAKYEFRSPDC-FKKLVITINGKTPGPSIQAQE

AtAAO3 ALFF---------SSVLCQGKIRRFKWEVKYEFKSPDC-FEKLVITINGKFPGPTIKAQQ

BrAAO5 TLFS---------SSVLVQGKIRRFKWEVKYELKSPDC-FEKLVITINGQLPGPTIKAQQ

HvAAO2 VASA---------LAAVAQAKTVHEHWDISYQFTHSDC-VRKLAVTINGGTPGPTIRAVQ

TaAAO5 -LWA---------LAAVAEAKTVHKQWDISYQFTHSDC-VRKLAVTINGRTPGPTIRAVQ

TaAAO1 FLWA---------LAAVAEAKTVHEQWDISYQFTHSDC-VRKLAVTINGGTPGPTIRAVQ

TaAAO3 -LWV---------LAAVAEAKTVHEQWEISYQFTHSDC-VRKLAVTINGGTPGPTIRAVQ

OsAAO4 AAAL---------FGV-AAAATRRHDWDISYQFTSPDC-VRKLAVTINGHTPGPTIRAVQ

ZmAAO2 CLWL---------STL-ARAATRRYEWEVSYQFKSPDC-VRKLSATINGQTPGPTIRATQ

SbAAO3 CLSL---------STLLARAATRRHEWEVSYQFKSPDC-VRKLSATINGQTPGPTIRATQ

ZmAAO1 SLSL---------AAVVARAATRHQEWEISYQFKSPDC-VRKLAVTINGETPGPTIRATQ

SbAAO2 SLSL---------AAAARA-ATVHQEWEISYQFKSPDC-VRKLAVTINGQTPGPTIRATQ

SbAAO1 SLSL---------AAAARAAATVHQEWEISYQFKSPDC-VRKLAVTINGQTPGPTIRATQ

ZmAAO3 SLAA---------LSVSADAKVHHHTWDIAYHYKSLDC-VNKLAVTINGESPGPTIRATQ

SbAAO4 ALAA---------LSVYAEAKVHHYTWDIAYHYKSLDC-VEKLAVTINGESPGPTIHATQ

OsAAO5 AA-A---------AAAPAEAKTHHHTWNITYQYKSPDC-FRKLAVTINGESPGPTIRAAQ

HvAAO4 SS-L---------LLAVADARVHNYTWDISYQYKSPDC-FEKLAVTVNGEAPGPTIRATQ

TaAAO6.1 FW-L---------LVAVAEAKVHHYKWDISYQLKSPDC-FEKLAVTVNGEAPGPTIRATL

TaAAO2 FW-L---------LVAVAEAKVHHYTWDISYQLKSPDC-FEKLAVTVNGEAPGPTIRATQ

TaAAO4.1 FW-L---------LVAVAEAKVHHYTWDISYQLKSPDC-FEKLAVTVNGEAPGPTIRATL

AtAAO2.1 VLVVAV-------LTHTASAAVREYHWEVEYKYWSPDC-KEGAVMTVNGEFPGPTIKAFA

BrAAO3 VV-VAL-------LAHTASAAVREYHWEVEYKFGFPDC-KEGMVMAVNGQFPGPTIHALA

BrAAO4 VA-VAI-------LAHTASAAVREYAWEVEYKFGWPDC-KEGMVMAVNGQFPGPTIHALA

AtAAO1 LTVVVV-------AFHSASAAVVESTWEVEYKYWWPDC-KEGIVMAINGQFPGPTIDAVA

BrAAO6 VTVVLV-------AVHSASARVVELDWEVEYKFWWPDC-KEGIVMAINGQFPGPTIDAVA

BrAAO1 VTVVMV-------AVHSASAGVVELNWEVEYKLRWPDC-KEGIVIAINGEFPGPTIDATA

BrAAO2 ------------------------------------------------------------

TaAAO8.1 ACGGLL-------HCA-EAAKARHLKWEVGHMFWSPDC-EEKVLIGINGQFPGPTIRAKA

HvAAO1 ACGGLL-------HCA-EAAKARHLKWEVSHMFWSPDC-EEKVLIGINGQFPGPTIRAKA

TaAAO9 ACAGVM-------HCT-EAAKARHLKWEVSHMFWSPDC-EEKVLIGINGQFPGPTIRAKA

TaAAO11 ACGGVM-------HCA-EAAKARHLKWEVSHMFWSPDC-EEKVLIGINGQFPGPTIRAKA

OsAAO1 ---AMS-------QCA-AAAKARHFRWEVSNMFWSPDC-EEKVVIGINGQFPGPTIRAKA

ZmAAO4 ---ARQ-------HCAA-AGKARHLRWEISNMFWSPDC-EEKVVIGINGQFPGPTIRARA

SbAAO5 ---AWQ-------HCAAEAAKARHFKWEISNMFWSPDC-EEKVVIGINGQFPGPTIRARA

CsAAO FGIT---------LSEAGFPKIKHYKWDVEYMFWSPDC-VENIVMGINGEFPGPTIRANA

CpAAO -------------------SQIRHYKWEVEYMFWAPNC-NENIVMGINGQFPGPTIRANA

CmAAO FGIS---------SE---GSQIRHYKWEVEYMFWAPDC-NENIVMGINGQFPGPTIRANA

NtAAO1 ELSS---------SRSVMAAKTRHFKWDVEYIHWSPDG-EESVVMGINGQFPGPTIRAKA

GmAAO4 -LSL---------LQLSLGAVRHRIRFNVEYMYREPDC-HEHVVMGINGQFPGPTITAEA

GmAAO7.1 -LGL---------VELSLGGRVRHYKFDVEYMIRKPDC-LEHVVMGINGQFPGPTIRAEV

GmAAO6 -LGL---------VELSIGGIVRHYKFDVEYMIRKPDC-LEHVLMGINGQFPGPTIRAEV

GmAAO3 -LAF---------AQLSLGGRVRHYKFDVEYMIRKPDC-LEHVVMGINGQFPGPTIRAEV

GmAAO5 -LGL---------AHLSLGGRVRHYKFDVEYMIRKPDC-LEHVVMGINGQFPGPTIRAEV

SbAAO6 MPVSAQSVAAAPS-PSSPAPATRNFTWDVEYIMWAPDC-HQSVMIGINGEFPGPTISANA

OsAAO2 VVAAAAAMAAACCAGMAAAAATVEVTWDVEYVLWAPDC-QQRVMIGINGRFPGPNITARA

HvAAO3 LAFRAPATTAASAPAPAPTPTTLNMTWDVEYILWAPDC-QQRVMIGINGKFPGPNITARA

TaAAO7 LAFAAPATTAASAPAPAPTPAKQTMTWDVEYIMWAPDC-QQRVMIGINGKFPGPNITARA

TaAAO10 LAAAAPATTAASVPAPAPTPTKQNMTWDVEYIMWSPDC-QQRVMIGINGKFPGPNITARA

TaAAO12 LALAAPATTAASVPAPALTPAKQNMTWDVEYILWSPDC-QQRVMIGINGKFPGPNITARA

NtAAO2 NNNIVVNVFNNLD-EPFLFTWNGVQHRKNSWQDGTPG-TMCPIMPGQNFTYRFQVKDQIG

BnAAO NNNVIINVFNNLD-EPFLLTWNGIQHRKNCWQDGTPG-TMCPIMPGTNYTYHFQPKDQIG

OsAAO3 NDNIIVNVFNNLD-EPFLLTWNGIKQRKNSWQDGVLG-TNCPIPPGANYTYKFQAKDQIG

GmAAO1 GDTIVVQVNNSLVTENLSIHWHGIRQIGTPWFDGTEGVTQCPILPGDTFIYQFVV-DRPG

GmAAO2 GDTIIVQVNNSLVTENLSIHWHGIRQIGTPWFDGTEGVTQCPILPGDTFIYQFVV-DRPG

AtAAO3 GDTIVVELKNSFMTENVAVHWHGIRQIGTPWFDGVEGVTQCPILPGEVFIYQFVV-DRPG

BrAAO5 GDTIIVELKNSFMTENVAVHWHGIRQVGTPWFDGVEGVTQCPILPGEIFTYQFVV-DRPG

HvAAO2 GDTVVVTVKNLLMTENVAIHWHGIRQLGTPWADGTEGVTQCPILPGDTFEYRFVV-DRPG

TaAAO5 GDTVVVTVKNLLMTENVAIHWHGIRQLGTPWADGTEGVTQCPILPGDAFEYRFVV-DRPG

TaAAO1 GDTVVVTVKNLLMTENVAIHWHGIRQLGTPWADGTEGVTQCPILPGDTFEYRFVV-DRPG

TaAAO3 GDTVVVTVKNLLMTENVAIHWHGIRQLGTPWADGTEGVTQCPILPGDTFEYRFVV-DRPG

OsAAO4 GDTIVVNVKNSLLTENVAIHWHGIRQIGTPWADGTEGVTQCPILPGDTFAYTFVV-DRPG

ZmAAO2 GDTVEVKVRNSLLTENLAIHWHGIRQIGTPWADGTEGVTQCPILPGDTLTYAFVV-DRPG

SbAAO3 GDTVEVKVTNSLLTENLAIHWHGIRQIGTPWADGTEGVTQCPILPGDTFTYAFVV-DRPG

ZmAAO1 GDTVVVRVKNSLLTENVAIHWHGIRQRGTPWADGTEGVTQCPILPGDTFTYAFVV-DRPG

SbAAO2 GDTVVVRVKNSLLTENVAIHWHGIRQIGTPWADGTEGVTQCPILPGDTFTYTFVV-DRPG

SbAAO1 GDTVVVTVRNSLLTENVAIHWHGIRQIGTPWADGTEGVTQCPILPGDTFNYTFVV-DRPG

ZmAAO3 GDTVVVTVRNSLETENTGIHWHGIRQVGSPWADGTVGVTQCPILPGDTFTYRFVV-DRPG

SbAAO4 GDTVVVTVHNKLETENTGIHWHGIRQIGSPWADGTVGVTQCPILPGDTFTYRFVV-DRPG

OsAAO5 GDTLVVTVHNMLDTENTAIHWHGIRQIGSPWADGTAGVTQCPILPGETFTYRFVV-DRPG

HvAAO4 GDTIVVNVHNKLETENTAIHWHGIRQIDTPWADGVAGVTQCPILPGETFAYKFVV-DRPG

TaAAO6.1 GDTIVVDVHNKLETENTAIHWHGIRQIGTPWADGVAGVTQCPILPGETFTYKFVI-DRPG

TaAAO2 GDTIVVAVHNKLETENTAIHWHGIRQIDTPWADGVAGVTQCPILPGETFTYQFVV-DRPG

TaAAO4.1 GDTIIVAVHNKLETENTAIHWHGIRQIDTPWADGVAGVTQCPILPGETFTYKFVV-DRPG

AtAAO2.1 GDTIVVNLTNKLTTEGLVIHWHGIRQFGSPWADGAAGVTQCAINPGETFTYNFTV-EKPG

BrAAO3 GDTIVVHLTNKLATEGLVIHWHGIRQLGSPWADGAAGVTQCAITPGETFTYKFTV-DKPG

BrAAO4 GDTIVVHLTNKLATEGLVIHWHGIRQLGSPWADGAAGVTQCAISPGETFTYNFTV-DKPG

AtAAO1 GDTVIIHVVNKLSTEGVVIHWHGIRQKGTPWADGAAGVTQCPINPGETFTYKFIV-DKAG

BrAAO6 GDIVIIHLTNKLSTEGVVIHWHGIRQRGTPWSDGAAGVTQCPINPGETFTYNFTV-DKPG

BrAAO1 GDTFIIHVTNKLSTEGVVIHWHGIRQNGTPWADGAAGVTQCPINPGETFTYNFIV-DKAG

BrAAO2 ------------------------------------------------------M-VQAG

TaAAO8.1 GDTIVVELKNGLHTEGVVIHWHGVRQIGTPWADGTAAISQCAINPEETFTYRFVV-DKPG

HvAAO1 GDTIVVELKNGLHTEGVVIHWHGVRQIGTPWADGTAAISQCAINPEETFTYRFVV-DKPG

TaAAO9 GDTIVVELKNGLHTEGVVIHWHGVRQIGTPWADGTAAISQCAINPEETFTYRFVV-DKPG

TaAAO11 GDTIVVELKNGLHTEGVVIHWHGVRQIGTPWADGTAAISQCAINPEETFTYRFVV-DKPG

OsAAO1 GDTIVVHLKNGLHTEGVVIHWHGIRQIGTPWADGTASISQCAINPEETFTYRFVV-DKPG

ZmAAO4 GDTVHVQLRNALHTEGVVIHWHGIRQIGTPWADGTAAISQCAINPEETFTYRFVV-DKPG

SbAAO5 GDTIHVQLKNALHTEGVVIHWHGIRQIGTPWADGTAAISQCAINPEETFTYRFVV-DKPG

CsAAO GDIVVVELTNKLHTEGVVIHWHGILQRGTPWADGTASISQCAINPGETFTYRFVV-DKAG

CpAAO GDSVVVELTNKLHTEGVVIHWHGILQRGTPWADGTASISQCAINPGETFFYNFTV-DNPG

CmAAO GDTVVVELINKLHTEGVVIHWHGILQRGTPWADGTASISQCAINPGETFFYNFTV-DNPG

NtAAO1 GDTVAVHLTNKLHTEGVVIHWHGIRQIGTPWADGTAAISQCAINPGETFLYRFKV-DKAG

GmAAO4 GDTLEILLTNKLSTEGTVIHWHGIRQYGTPWADGTAAISQCAIAPGETFNYTFTV-DRPG

GmAAO7.1 GDILDIALTNKLFSEGTVVHWHGIRQVGTPWADGTASISQCAINPGETYHYRFTV-DRPG

GmAAO6 GDILDIALTNKLFTEGTVIHWHGIRQVGTPWADGTASISQCAINPGETFHYKFTV-DRPG

GmAAO3 GDILDIALTNKLFTEGTVIHWHGIRQVGTPWADGTAAISQCAINPGETFQYRFTV-DRPG

GmAAO5 GDILDIALTNKLFTEGTVIHWHGIRQVGTPWADGTAAISQCAINPGEAFHYRFTV-DRPG

SbAAO6 GDLIRVEVTNSLHTEGVVIHWHGIRQIGTPWADGTASISQCPINSGERFTYEFIA-DKPG

OsAAO2 GDVISVTMNNKMHTEGVVIHWHGIRQFGTPWADGTASISQCAVNPGETFVYKFVA-DKPG

HvAAO3 GETVSITVNNKLHTEGLVIHWHGMRQVGTPWADGTASISQCAVSPGESFTYEFVA-DKPG

TaAAO7 GETLSITVNNKLHTEGLVIHWHGMRQVGTPWADGTASISQCAVSPGDSFTYEFVA-DKPG

TaAAO10 GETLSITVNNKLHTEGLVIHWHGMRQVGTPWADGTASISQCAISPGDSFTYEFVA-DKPG

TaAAO12 GETLSITVNNKLHTEGLVIHWHGMRQVGTPWADGTASISQCAISPGDSFTYEFVA-DKPG

. *

NtAAO2 SYSYFPTTALHRAAGGYGALNVHSRAL------IPVPFDNPAD----EYNVFVGDWYNKG

BnAAO SYFYYPTTGMHRAAGGYGGLRVNSRLL------IPVPYADPED----DYTVLIGDWYTKS

OsAAO3 TFVYFPSVAMHRAAGGFGALNVYQRPA------IPVPYPPPAG----DFTLLVGDWYKAG

GmAAO1 TYLYHAHYGMQREAGLYGMIRVAPR--------DPEPFA--YDL---DRSIILNDWYHKS

GmAAO2 TYLYHAHYGIQREAGLYGMMRVAPR--------DPEPFA--YDL---DRSIILNDWYHSS

AtAAO3 TYMYHSHYGMQRESGLIGMIQVSPP------ATEPEPFT--YDY---DRNFLLTDWYHKS

BrAAO5 TYMYHSHYGMQRESGLIGMIRVSPP------STEPEPFT--YDY---DRSLLLTDWYHKG

HvAAO2 TYMYHAHYGMQRSAGLNGMIVVAAAPG----GPDAEPFA--YDGG--EHDVLLNDWWHKS

TaAAO5 TYMYHAHYGMQRSAGLNGMIVVAAAPG----SADAEPFA--YDGG--EHEVLLNDWWHKS

TaAAO1 TYMYHAHYGMQRSAGLNGMIVVAAAPG----SADAEPFA--YDGG--EHEVLLNDWWHKS

TaAAO3 TYMYHAHYGMQRSAGLNGMIVVAAAPG----GADAEPFA--YDGG--EHDVLLNDWWHKS

OsAAO4 TYMYHAHYGMQRSAGLNGMIVVEVAPGAA-GDGEREPFR--YDG---EHTVLLNDWWHRS

ZmAAO2 TYMYHAHYGMQRSAGLYGVVVVVVAAAPGAKADDAEPFA--YDD---EHHVLLNDWWHNS

SbAAO3 TYMYHAHYGMQRSAGLYGVIVVAVAEDPGGEVDGAEPFA--YDD---EHDVLLNDWWHSS

ZmAAO1 TYMYHAHYGMQRSAGLNGLIVVAAA--PG--GPDAEPFR--YDG---EHDVLLNDWWHKS

SbAAO2 TYMYHAHYGMQRSAGLNGLIVVAAA--RG--GPDAEPFR--YDG---EHHVLLNDWWHKS

SbAAO1 TYMYHAHYGMQRSAGLNGMIVVNSA--RG--GPDGEPFT--YDG---EHDVLLNDWWHKS

ZmAAO3 TYFYHAHYGMQRVAGLDGMLVVSV---PDG---VAEPFA--YDE---DRTVLLMDWWHKS

SbAAO4 TYFYHAHYGMQRVAGLDGMLVVSSSSVPDG-GVAAEPFT--YDE---ERTVLLMDWWHKS

OsAAO5 TYMYHAHYGMQRVAGLDGMLVVSV---PDG---VAEPFA--YDG---EHTVLLMDWWHQS

HvAAO4 TYLYHAHYGMQRVAGLNGMIVVKV---PDG---IVEPFS--YDE---EHTVLLGDWWHKS

TaAAO6.1 TYLYHAHYGMQRVAGLNGMIVVTV---PEG---FVEPFS--YDE---EHTVLLGDWWHKS

TaAAO2 TYLYHAHYGMQRVAGLNGMIVVTV---PDG---FVEPFS--YDE---EHTVLLGDWWHKS

TaAAO4.1 TYLYHAHYGMQRVAGLNGMIVVTV---PDG---FVEPFS--YDE---EHTVLLGDWWHKS

AtAAO2.1 THFYHGHYGMQRSAGLYGSLIVDVAKG------KSERLR--YDGE--F-NLLLSDWWHEA

BrAAO3 THFYHGHYGMQRSAGLYGSLIIDAAKG------KIEPLR--YDGE--F-NLLLSDWWHES

BrAAO4 THFYHGHYGMQRSAGLYGSLIIDVAKG------KKEPLR--YDGE--F-NLLLSDWWHED

AtAAO1 THFYHGHYGMQRSSGLYGMLIVRS---------PKERLI--YDGE--F-NLLLSDWWHQS

BrAAO6 THFYHGHYGMQRSAGLYGMMIVRS---------PKEKLR--YDGE--F-NLLLSDWWHQS

BrAAO1 THFYHGHYGMQRSAGLYGMMIVRS---------PKETLQ--YDGE--F-NLLLSDWWHQG

BrAAO2 THFYHGHYGMQRSAGLYGMMVVRS---------PKETLQ--YDGE--F-NLLLSDWWHLS

TaAAO8.1 TYFYHGHYGMQRAAGLYGSLIVDVADG------EEEPFK--YDGE--L-NLLLSDWYHDS

HvAAO1 TYFYHGHYGMQRAAGLYGSLIVDVADG------EEEPFK--YDGE--L-NLLLSDWYHES

TaAAO9 TYFYHGHYGMQRAAGLYGSLIVDVADG------EEEPFK--YDGE--L-NLLLSDWYHDS

TaAAO11 TYFYHGHYGMQRAAGLYGSLIVDVADG------EEEPFK--YDGE--L-NLLLSDWYHES

OsAAO1 TYFYHGHYGMQRAAGLYGSLIVDVADG------DEEPFK--YDGE--I-NLLLSDWYHES

ZmAAO4 TYFYHGHYGMQRAAGLYGSLVVDVAEG------EEEPFQ--YDGE--L-NLLLSDWYHES

SbAAO5 TYFYHGHYGMQRAAGLYGSLIVDVAEG------EEEPFK--YDGE--L-NLLLSDWYHES

CsAAO TYFYHGHLGMQRSAGLYGSLIVDPPEG------RSEPFH--YDEE--I-NLLLSDWWHQS

CpAAO TFFYHGHLGMQRSAGLYGSLIVDPPQG------KKEPFH--YDGE--I-NLLLSDWWHQS

CmAAO TFFYHGHLGMQRSAGLYGSLIVDPPQG------KKEPFH--YDGE--I-NLLLSDWWHQS

NtAAO1 TYFYHGHYGMQRSAGLYGSLIVEVGEG------EKEPFH--YDGE--F-NLLLSDWWHKG

GmAAO4 TYFYHGHFGMQRAAGLYGSLIVNLPKG------KKEPFH--YDGE--F-NLLLSDWWHKS

GmAAO7.1 TYFYHGHYGMQRAAGLD----------------KTNRFH--YDGE--F-NLLLSDLWHTS

GmAAO6 TYFYHGHHGMQRAAGLYGSLIVDLPKG------QNEPFH--YDGE--F-NLLFSDLWHTS

GmAAO3 TYFYHGHHGMQRSAGLYGSLIVDLPKG------QNEPFP--YDGE--F-NLLLSDLWHTS

GmAAO5 TYFYHGHHGMQRSAGLYGSLIVDLPKG------QNEPFH--YDGE--F-NLLLSDLWHTS

SbAAO6 TFFYHGHFGMQRAAGLYGSLIVNGTEQ------QPEPFAAEYDGE--L-NMLLSDWYHEN

OsAAO2 TYFYHGHFGMQRAAGLYGSLIVLDSPE------QPEPFRHQYDDGGELPMMLLSDWWHQN

HvAAO3 TYFYHGHFGMQRAAGLYGWLVVDATAE------QGEPYRSDYDGG-EL-RMLLSDWYHES

TaAAO7 TYFYHGHFGMQRAAGLYGWLVVDATAE------RGEPYRRDYDGG-EL-RMLLSDWYHDN

TaAAO10 TYFYHGHFGMQRAAGLYGWLVVNATAE------QDEPYRRDYDGG-EL-RMLLSDWYHDN

TaAAO12 TYFYHGHFGMQRAAGLYGWLVVNATAE------QDEPYRRDYDGG-EL-RMLLSDWYHDN

:. *. .::* :* . .:. * :

NtAAO2 HKTLKKILDG----GRTIGRPDGIIINGKSAKV---------------------------

BnAAO HTQLKKFLDG----GRTIGRPDGIVINGKSGKG---------------------------

OsAAO3 HKQLRQALDA--GGGGALPPPDALLINGMPS-----------------------------

GmAAO1 TYEQAAGLSS--IPFQWVGEPQSLLIHGKGRFN---CSKS----PS--------------

GmAAO2 TYEQAAGLSS--IPFRWVGEPQSLLIHGKGIFN---CSKS----PS--------------

AtAAO3 MSEKATGLAS--IPFKWVGEPQSLMIQGRGRFN---CSNNLTTPPS--------------

BrAAO5 MSEKATGLAS--IPFKWVGEPQSLMIQGRGRFN---CTNNMMTPQR--------------

HvAAO2 TYEQAAGLAA--VPIEWVGEPKSLLINGRGRYN--CSAMAS--D----------------

TaAAO5 TYEQAAGLAA--VPIVWVGEPQSLLINGRGRYN--CSAMAP--D----------------

TaAAO1 TYEQAAGLAA--VPIVWVGEPQSLLINGRGRYN--CSAMAS--D----------------

TaAAO3 TYEQAAGLAA--VPIVWVGEPQSLLINGRGRYN--CSAMAP--D----------------

OsAAO4 TYEQAAGLAS--VPMVWVGEPQSLLINGRGRFV--NCSSSP--A----------------

ZmAAO2 TYELAVGLAS--VPMVWVGEPHSLLINGRGRFN---CSA-A--A----------------

SbAAO3 THEQAVGLAS--VPMVWVGEPHSLLINGRGRFD---CSA-A--A----------------

ZmAAO1 TYEQAAGLAS--APLVWVGEPQSLLINGRGRFV--NCSAA--------------------

SbAAO2 TYEQATGLAS--VPLGWVGEPQSLLINGRGRFV--NCSAAA--A----------------

SbAAO1 TYEEATGLAS--VPIIWVGEPQSLLINGRGRWF--SCSRMA--A----------------

ZmAAO3 VYEQAVGLAS--DPLVFVGEPQSLLINGRGVFEPFHCSRAP--SAS--------------

SbAAO4 VYEQAVGLAS--DPLRFVGEPQSLLINGRGVFEPFHCSRAP--NGS--------------

OsAAO5 VYEQAVGLAS--VPMVFVGEPQSLLINGRGVF---NCSPPA--ASN--------------

HvAAO4 VYEQATGLTE--TPFVFVTAPQSLLINGRGTF---DCSLAP-------------------

TaAAO6.1 VYEQATGLSA--NPFVFVTEPQSLLINGRGMF---NCSLAP-------------------

TaAAO2 VYEQATGLSS--NPFVFVTEPQSLLINGRGMF---NCSLAP-------------------

TaAAO4.1 VYEQATGLSS--NPFVFVTEPQSLLINGRGMF---NCSLAP-------------------

AtAAO2.1 IPSQELGLSS--KPMRWIGEAQSILINGRGQFN---CSLAAQFSN---------------

BrAAO3 VLSQELGLSA--KPMRWIGEAQSILINGRGQFK---CSLAAQFSNATA------------

BrAAO4 VLSQEIGLSS--RPMRWIGEAQSILINGRGQFN---CSLAAQF-----------------

AtAAO1 IHAQELALSS--RPMRWIGEPQSLLINGRGQFN---CSQAAYFNKG--------------

BrAAO6 THSQELALSS--SPMRWIGEPQSLLINGRGQFN---CSQAAYLSNE--------------

BrAAO1 SHAQELYLSS--RPMRWIGEPQSLLINGRGQFN---CSLAAYFNEG--------------

BrAAO2 STLQELSLSS--KPMRWIGEPQSLLINGRGQFD---CSQAGYFNEG--------------

TaAAO8.1 IYNQMVGLSS--SPMRWIGEPQSLLINGRGQFN---CSLAAAHTPG--------------

HvAAO1 IYNQMVGLSS--SPMRWIGEPQSLLINGRGQFN---CSLAAAHTPG--------------

TaAAO9 IYNQMVGLSS--SPMRWIGEPQSLLINGRGQFN---CSLAAAHTPG--------------

TaAAO11 IYNQMVGLSS--SPMRWIGEPQSLLINGRGQFN---CSLAAAHTPG--------------

OsAAO1 IYTQMVGLSS--NPFRWIGEPQSLLINGRGQFN---CSLAAAHTPG--------------

ZmAAO4 IHTQMVALSS--RPFRWIGEPQSLLINGRGQFN---CSLAAAHTQG--------------

SbAAO5 IHTQMVALSS--KPFRWIGEPQSLLINGRGQFN---CSLAAAHTPG--------------

CsAAO VHKQEVGLSS--KPMRWIGEPQSILINGKGQFD---CSIAAKYN-Q--------------

CpAAO IHKQEVGLSS--KPIRWIGEPQTILLNGRGQFD---CSIAAKYD-S--------------

CmAAO IHKQEVGLSS--KPIRWIGEPQTILLNGRGQFD---CSIAAKYD-S--------------

NtAAO1 SHEQEVDLSS--NPLRWIGEPQTLLLNGRGQYN---CSLAARFSKP--------------

GmAAO4 THSQEVGLSS--MPFRWINEPQSLLINGRGQYN---CSLAASLIKT--------------

GmAAO7.1 SHEQEVGLST--KPLKWIGEPQTLLINGRGQFN---CSLASKFINT--------------

GmAAO6 SHEQEVGLST--KPLKWIGEPQTLLINGRGQFN---CSLASKFINT--------------

GmAAO3 SHEQEVGLSS--KPFKWIGEAQTLLINGRGQFN---CSLASKFINT--------------

GmAAO5 SHEQEVGLSS--KPFKWIGEPQTLLINGKGQFN---CSLASKFINT--------------

SbAAO6 VYAQAAGLDGKDKHWEWVGEPQTILINGRGQFG---CSLGITGD---------RRACDRR

OsAAO2 VYAQAAGLDGKDRHFEWIGEPQTILINGRGQFE---CTLGPARKSFEKLLNENVETCVD-

HvAAO3 VYAQAAGLERKDKHFEWVGEPQTILINGRGQHD---CMLGAVTG-YHRGIDRRARTCVRG

TaAAO7 VYAQAAGLEQKYDHFQWVGEPQTILVNGRGQYD---CMLGAVTR-FHRGIDRRARTCVRG

TaAAO10 VYAQAAGLEQKYDHFQWVGEPQTILINGRGQHD---CMLGTVTR-FHRGIDRHAKTCVRD

TaAAO12 VYAQAAGLEQKYDHFQWVGEPQTILINGRGQYD---CMLGAVTR-FHRGIDRRARTCVRG

* : . ::::*

NtAAO2 -----------------GEA-------------KEPLFTMEAGKTYRYRFCNLGMRSSVN

BnAAO -----------------DGS-------------DAPLFTLKPGKTYRVRICNVGVKTSIN

OsAAO3 ----------------------------------AAAFVGDQGRTYLFRVSNVGVKTSVN

GmAAO1 -----------------VSTDVC--DTSNPQCSPF-VQTVIPGKTYRLRIASLTALSALS

GmAAO2 -----------------LGTDVC--DAS--KCSPF-VQTVIPGKTYRLRIASLTALSALS

AtAAO3 -----------------LVSGVC--NVSNADCSRF-ILTVIPGKTYRLRIGSLTALSALS

BrAAO5 -----------------SEAEVC--NASHADCSRF-VLMVIPGKTYRLRIGSLTSLSALS

HvAAO2 ------------------AAAAC--NATHPECATQ-VFAVVPGKTYRFRIASVTSLSALN

TaAAO5 --------------------AAC--NATHPECAAQ-VFAVVPGRTYRFRIASVTSLSALN

TaAAO1 ------------------AAAAC--NATHPECAPQ-VFAVVPGRTYRFRIASVTSLSALN

TaAAO3 ------------------A-AAC--NATHPECAAQ-VFAVVPGRTYRFRIASVTSLSALN

OsAAO4 ------------------TAASC--NVSHPDCAPA-VFAVVPGKTYRFRVASVTSLSALN

ZmAAO2 ------------------VPGTC--NATSPECDTP-VFAVVPGQTYRFRIASVTSLSALN

SbAAO3 ------------------VPGTC--NATSPECAAP-VFAVVPGKTYRFRIASVTSLSALN

ZmAAO1 --------------------GAC--DAAHPECATP-VFAVVPGRTYRFRIASVTSLSALN

SbAAO2 ------------------AGMTC--NATLPECAAP-VFAVVPGKTYRFRIASVTSLSALN

SbAAO1 --------------------GAC--NATLPECAAP-VFAVVPGKTYRFRIASVTSLSALN

ZmAAO3 -----------------GCSSAP--RP-AAGCAPPALFTAVPGKTYRLRVGSLTSLSALN

SbAAO4 -----------------GDAACN--RPRPAGCAPPTLFTAVPRKTYRLRVGSLTSLSALN

OsAAO5 -----------------GGGAAC--NAFGGECGWPTLFTASPGKTYRLRIGSLTSLASLS

HvAAO4 -------------------SGTC--NASSPDCGLRPLFTAVPGKTYLLRIGSLTSLSSLS

TaAAO6.1 -------------------SGTC--NASRPDCALPTLFTAVPGKTYLLRIGSLTSLSSLY

TaAAO2 -------------------SGTC--NASRPDCALPTLFTAVPGKTYLLRIGSLTSLSSLY

TaAAO4.1 -------------------SGTC--NASRPDCALPTLFTAVPGKTYLLRIGSLTSLSSLY

AtAAO2.1 ----------------NTSLPMC-TFKEGDQCAPQ-ILHVEPNKTYRIRLSSTTALASLN

BrAAO3 -------------QFSNTSLPMC-KFKKGDQCAPQ-RLHVEPNKTYRIRLASTTGLASLN

BrAAO4 ---------------SSTSLPTC-TFKEGDQCAPQ-RLHVEPNKTYRIRLASSTALASLN

AtAAO1 -----------------GEKDVC-TFKENDQCAPQ-TLRVEPNRVYRLRIASTTALASLN

BrAAO6 -----------------GKR-QC-TFRKNDQCAPE-TLRVRPNKVYRLRIASTTALASLN

BrAAO1 -----------------GLK-EC-KFKDNDDCAPT-ILRVEPLKVYRLRIASTTALASLN

BrAAO2 -----------------GLK-EC-NFTKDDPCAPT-TLRVEPNKVYRLRIASTTSLASLN

TaAAO8.1 -----------------TKQ--C-TAGGNRHCAPV-ILPVQPNKTYRLRIASTTSLASLN

HvAAO1 -----------------TKQ--C-TAGGNRHCAPV-ILPVEPNKTYRLRIASTTSLASLN

TaAAO9 -----------------TKQ--C-TAGGNRHCAPV-ILPVEPNKTYRLRIASTTSLASLN

TaAAO11 -----------------TKQ--C-TAGGNRHCAPV-ILPVEPNKTYRLRIASTTSLASLN

OsAAO1 -----------------AKQ--C-AAAGNRHCAPV-ILPVLPNKTYRLRVASTTSLASLN

ZmAAO4 -----------------ATNTQCAATAANTQCAPV-VLPVQPNKTYRLRVASTTSLASLN

SbAAO5 -----------------A--NQCAAAAVNTQCAPV-VFPVQPNKTYRLRVASTTSLASLN

CsAAO ------------------GLKQC-ELSGKEKCAPF-ILHVQPKKTYRIRIASTTALASLN

CpAAO ------------------NLEPC-KLKGSESCAPY-IFHVSPKKTYRIRIASTTALAALN

CmAAO ------------------NLEPC-KLKGSEPCAPY-IFHVMPKKTYRIRIASTTALAALN

NtAAO1 ------------------PLPQC-KLRGGEQYAPQ-ILRVRPNKIYRLRVASTTALGSLS

GmAAO4 ------------------SLPQC-KFRGNEQCAPQ-ILHVDPNKTYRIRIASTTSLASLN

GmAAO7.1 ------------------TLPQC-HLKGDEECAPQ-ILDVEPNKTYRIRIASTTSLAALN

GmAAO6 ------------------TLPEC-QFKGGEECAPQ-ILHVEPNKTYRIRIASTTSLAALN

GmAAO3 ------------------TLPQC-QLKGGEECAPQ-ILHVEPNKTYRIRIASTTALASLN

GmAAO5 ------------------TLPQC-QLKGGEECAPQ-ILHVEPNKTYRIRIASTTALASLN

SbAAO6 KRDALCKEGDKSERCELIRRSECGPFCERSQCSPV-VFDVDPGKTYRLRIASTTSLSALN

OsAAO2 -DQKMCSDQE-----KCLRRSECGPYCPRSQCAPV-VFNVEQGKTYRLRIASTTSLSLLN

HvAAO3 KEAQLCRDEE-----RCLRRSECGPYCPESQCAPV-VFDVEPGRTYRLRIASTTSLSALN

TaAAO7 KEAKLCGDEE-----RCLRRSECGPYCPESQCAPV-VLDVEPGRTYRLRIASTTSLAALN

TaAAO10 KQAKLCRDEE-----RCLRRSECGPYCPQSQCYPV-VFDVEPGRTYRLRIASTTSLSALN

TaAAO12 KEAKLCGDEE-----RCLRRSECGPYCPQSQCAPV-VFDVEPGRTYRLRIASTTSLSALN

: * *. . :

NtAAO2 IRFQGHPMKLVELEGSHTVQNIYDSLDLHVGQCLSVLVTAD-QEP-----KDYYLVVSSR

BnAAO FRIQNHKMKLVEMEGSHVLQNDYDSLDVHVGQCFGTIVTAN-QEP-----KDYYMVASSR

OsAAO3 VRIQGHSLRLVEVEGTHPVQNVYDSLDVHVGQSVAFLVTLD-KAA-----QDYAVVASAR

GmAAO1 FEIEANDMTVVEADGHYVEPFEVKNLFIYSGETYSVLVKTD-QDP----SRNYWITSNVV

GmAAO2 FQIEGHNMTVVEADGHYVEPFVVKNLFIYSGETYSVTVKSD-QDP----SRNYWITSNVV

AtAAO3 FQIEGHNLTVVEADGHYVEPFTVKNLFVYSGETYSVLLKAD-QNP----RRNYWITSSIV

BrAAO5 FQIEGHNLTVVEADGHYVEPFTVRNLFIYSGETYSVLLKAD-QNP----SRNYWITTSIV

HvAAO2 FEIEGHEMTVVETDGHYVKPFVVKNLNIYSGETYSVLIKAD-QDP----NRNYWLASNVV

TaAAO5 FEIEGHEMTVVETDGHYVKPFVVKNLNIYSGETYSVLIKAD-QDP----NRNYWLASNVV

TaAAO1 FEIEGHEMTVVETDGHYVKPFVVKNLNVYSGETYSVLIKAD-QDP----NRNYWLASNVV

TaAAO3 FEIEGHEMTVVETDGHYVKPFVVKNLNIYSGETYSVLIKAD-QDP----NRNYWLASNVV

OsAAO4 FEIEGHEMTVVEADGHYVKPFVVKNLNIYSGETYSVLITAD-QDP----NRNYWLASNVV

ZmAAO2 FEIEGHEMTVVEADGHYVKPFVVKNINIYSGETYSVLIKAD-QDP----NRNYWLASNVV

SbAAO3 FEIEGHEMTVVEADGHYVKPFVVKNLNIYSGETYSVLIKAD-QDP----NRNYWLASNVV

ZmAAO1 FEIEGHEMTVVEADGHYVKPFVVKNLNIYSGETYSVLIKAD-QDP----NRNYWLASNVV

SbAAO2 FEIEGHEMTVVEADGHYVKPFVVKNLNIYSGETYSVLIKAD-QDP----NRNYWLASNVV

SbAAO1 FEIEGHNMTVVEADGHYVKPFVVKNLNIYSGETYSVTITAD-QDP----NRNYWVASNVI

ZmAAO3 FAIEGHSMTVVEADGHYVRPVVVDSLYIYSGESYSVLLTAD-QDP----SRNYWAASHVV

SbAAO4 FAIEGHSMTVVEADGHYVRPVVVDSLYIYSGESYSVLVEAD-QDP----SRNYWAASHVV

OsAAO5 FEIEGHTMTVVEADGYYVTPVVVKNLFIYSGETYSVLVTAD-QDP----SRSYWAASHVV

HvAAO4 FEIEGHSMTVVEADGYYVRPFVVKNLFIYSGETYSVLVKAD-QDP----RRNYWAASHVV

TaAAO6.1 FEIEGHPMMVVEADGHYVRPFAVRGLFIYSGETYSVLVKAD-QDP----RRNYWAASHVV

TaAAO2 FEIEGHSMMVVEADGHYVLPFAVRGLFIYSGETYSVLVKAD-QDP----RRNYWAASHVV

TaAAO4.1 FEIEGHSMMVVEADGHYVRPFAVRGLFIYSGETYSVLVKAD-QDP----RRNYWAASHVV

AtAAO2.1 LAVQGHKLVVVEADGNYITPFTTDDIDIYSGESYSVLLTTD-QDP----SQNYYISVGVR

BrAAO3 FAVQGHKLVVVEADGNYITPFATSDVDIYSGESYSVLLTTD-QDP----SQNYWISVGVR

BrAAO4 FAVQGHKLVVVEADGNYITPFTTDDIDIYSGETYSVLLTTD-QDP----SQNYYITAGVR

AtAAO1 LAVQGHQLVVVEADGNYVAPFTVNDIDVYSGETYSVLLKTN-ALP----SKKYWISVGVR

BrAAO6 LAVEGHKLEVVEADGNYVKPFTVDDIDIYSGETYSVLLRTHS-FP----TKKHFISVGVR

BrAAO1 LAVEGHNLEVVEADGNYIAPFTVNDIDIYSGETYSVLLRTHNPTP----SRKYWISVGVR

BrAAO2 LAVEGHQLEVVEADGNYVAPFTVDDIDIYSGETYSVLLRTHNPSP----PRKYWISVGVR

TaAAO8.1 LAIGNHKLTVVEADGNYVEPFVVDDMDIYS------------------------------

HvAAO1 LAIGNHKLTVVEADGNYVEPFVVDDMDIYSGDSYSVLLTTD-QDP----SSNYWVSIGVR

TaAAO9 LAIGNHKLTVVEADGNYVEPFVVDDMDIYSGDSYSVLLTTD-QDP----SSNYWVSIGVR

TaAAO11 LAIGKHKLTVVEADGNYVAPFVVDDMDIYSGDSYSVLLTTD-QDP----SSNYWVSIGVR

OsAAO1 LAVGNHKLTVVEADGNYVEPFAVDDIDIYSGDSYSVLLTTD-QDT----SANYWVSVGVR

ZmAAO4 LAIGNHKLTVVEADGNYVDPFVVDDIDLYSGDSYSVLLTTD-QDT----SSNYWVSVGVR

SbAAO5 LAVGNHKLTVVEADGNYVDPFVVDDVDLYSGDSYSVLLTTD-QDT----SSNYWVSVGVR

CsAAO FAIGNHELLVVEADGNYVQPFVTSDIDIYSGESYSVLITTD-QNP----LENYWVSIGVR

CpAAO FAIGNHQLLVVEADGNYVQPFYTSDIDIYSGESYSVLITTD-QNP----SENYWVSVGTR

CmAAO FAIGNHPLLVVEADGNYVQPFYTSDIDIYSGESYSVLITTD-QNP----SENYWVSVGTR

NtAAO1 LAIGGHKMVVVEADGNYVQPFSVQDMDIYSGESYSVLFKTD-QDP----TKNYWISINVR

GmAAO4 LAIGDHKLVVVEADGNYVKPFIVDDIDIYSGESYSVLLTTN-QDP----KKNYWISVGVR

GmAAO7.1 LAISNHKLVVVEVDGNYVTPFAVDDMDIYSGESYSVLLHTN-QNP----NKNYWLSIGVR

GmAAO6 LAISNHKLVVVEADGNYVTPFAVDDVDIYSGESYSVLLRTD-QDP----NKNYWLSIGVR

GmAAO3 LAISNHKLVVVEADGNYVTPFAVDDIDIYSGESYSVLLRTD-QDP----NKNYWLSIGVR

GmAAO5 LAISNHKLVVVEADGNYVSPFAVDDIDIYSGESYSVLLRTD-QDP----NKNYWLSIGVR

SbAAO6 VQVQGHKLTVVEADGNFVAPFDVDDIDIYSGETYSVLLTTD-QRA---SSSSYWISVGVR

OsAAO2 VKIQGHKMTVVEADGNHVEPFVVDDIDIYSGESYSVLLKAD-QKP-----ASYWISVGVR

HvAAO3 VQVQGHELTVVEADGNPVEPFTVTDIDIYSGESYSVLLTTN-HTP-----GSFWVSVGVR

TaAAO7 VQVQGHELTVVEADGNPVEPFTVADIDIYSGESYSVLLTTN-HTPTFYRSGSFWVSVGVR

TaAAO10 VQVQGHDLTVVEADGNPVEPFTVPDIDIYSGESYSVLLTTN-HTPTFYRSGSFWVSVGVR

TaAAO12 VQVQGHDLTVVEADGNPVEPFTVPDIDIYSGESYSVLLTTN-HTPTFYRSGSFWISVGVR

. . : : :** :* .: ::

NtAAO2 FL-KQA--LSSVAIIRYANGKGPASP-ELPTPPPENTEGIAWSMNQFRSFRWNLTAS-AA

BnAAO FL-KTV--ITTTGLLRYEGGKGPASS-QLPAGP----VGWAWSLNQFRSFRWNLTAS-AA

OsAAO3 FS-PGASPLMATGTLHYSSAVSRAPG-PLPAPPPEQ---AEWSMNQARSFRWNLTAS-AA

GmAAO1 SR-NRTTP-PGLGMFNYYP---NHPK-RSPPTVPP--SPPAWDDVEPR-LAQSLSIK-AR

GmAAO2 SR-NRSTP-AGLGMFNYYP---NHPK-RSPPTVPP--SPPAWHDVEPR-LAQSFSIK-AR

AtAAO3 SR-PATTP-PATAVLNYYP---NHPR-RRPPTSESSNIVPEWNDTRSR-LAQSLAIK-AR

BrAAO5 SR-PEKTP-PATAVLNYHP---HHPR-KHPPTPASSNFRPEWNDTRHR-LAQSVAIK-AR

HvAAO2 SR-KPGTP-TGTAVLSYYGGR-SSPR-APPPTSPP--AGPAWDDTAYR-INQSLATV-AH

TaAAO5 SR-KPGTP-TGTAVLSYYGGR-SSPR-APPPTAPP--AGPAWNDSAYR-IRQSLATV-AH

TaAAO1 SR-KPGTP-TGTAVLSYYGGR-SSPR-APPPTAPP--AGPAWNDSAYR-INQSLATV-AH

TaAAO3 SR-KPGTP-TGTAVLSYYGGR-SSPR-APPPTAPP--AGPAWNDSAYR-IGQSLATV-AH

OsAAO4 SR-KPATP-TGTAVLAYYGGRRNSPR-ARPPTPPP--AGPAWNDTAYR-VRQSLATV-AH

ZmAAO2 SR-QPATP-TGTAILSYSGDR------APPSTTPP--TGPAWNDTMYR-FQQSVATV-AH

SbAAO3 SR-EPATP-TGTAILSYSGDR------APPSTTPP--TGPAWNDTMYR-FQQSVATV-AH

ZmAAO1 SR-EPGTP-AGTAVLSYYGGR-SSPR-RSPPTAPP--AGPAWNDTAYR-FRQSVATV-AH

SbAAO2 SR-EPGTP-TGTAVLSYYGGR-SSPR-KAPPTTPP--TGPAWNDTTYR-FRQSVATV-AH

SbAAO1 SR-EPGTP-TGTAILSYYSGR-SSPR-KPPPTTPP--TGPAWNDTTYR-FRQSVATV-AH

ZmAAO3 AR-ERNTT-SAMAI--L-SYAGNDPR-APPPTPRP--EGPAWDDARPR-LEQSRSLAVAH

SbAAO4 AR-ERNTT-SAVAI--L-SYASNDPR-AAPPTPRP--EGPAWDDVTAR-VEQSRSLAVAH

OsAAO5 SR-DPTKTAPGRAVVRY-ASAAV----DHPRTPPP--TGPRWNDTASR-VAQSRSFA-AL

HvAAO4 GR-NASQTPSGKAIVSY-AFKGNNPW-IPPPTAPP--AGPAWNNTVIR-VQQSRAIF-AH

TaAAO6.1 GR-NPSQTPSGKAVVSY-AFNGNNPW-MPPPTAPP--AGPPWNNTAIR-VDQSRAIF-AH

TaAAO2 GR-NPSQTPSGKAVVSY-AFNGNNPW-MPPPTAPP--AGRAWNNTAIR-VEQSRAIF-AH

TaAAO4.1 GR-NPSQTPTGKAVVSY-AFNGNNPW-MPPPTAPP--AGPPWNNTAIR-VEQSRAIF-AH

AtAAO2.1 GR-KPNTT-QALTILNYVTAPA----SKLPSSPPP--VTPRWDDFERS-KNFSKKIF-SA

BrAAO3 GR-KPKTP-PALTVLHYVTAPS----SQPPSSPPP--ETPRWNDFDRS-RNFSKRIF-SA

BrAAO4 GR-KPNTP-PALTVLNYVTAPS----SQLPTSPPP--ETPRWNDFDRS-KNFSKKIF-AA

AtAAO1 GR-EPKTP-QALTVINYVDATE----S-RPSHPPP--VTPIWNDTDRS-KSFSKKIF-AA

BrAAO6 GR-KPNTT-QALTVLSYIDAPE----SARPSLPPP--VTPRWDDYNRS-ISFSNKFF-AA

BrAAO1 GR-KPNTT-QALTLLHYADAPE----YEHLPYPPP--VTPRWDDYDRS-KTFSKKIF-AA

BrAAO2 GR-KPNTP-QALTMLHYAGASE----SERLPFPPR--ETPRWDDFNRS-KNFSKKIF-AA

TaAAO8.1 -------------------------------------VTPAWNDTAHS-KAFTTQIK-AR

HvAAO1 GR-APKTA-PALALLNYRPNRG----FKLPAIAPP--VTPAWNDTAHS-KAFTTQIK-AR

TaAAO9 GR-TPKTA-PALALLNYRPNRG----FKLPAIAPP--VTPLWNDTAHS-KAFTTHIK-AR

TaAAO11 GR-TPKTA-PALALLNYRPNRG----FKLPAIAPP--VTPAWNDTAHS-KAFTTQIK-AR

OsAAO1 GR-QPRTA-PALAVLNYRPNRA----SRLPAAAPP--ATPAWDDFARS-KAFTYRIL-GR

ZmAAO4 GR-LPKTA-PALAVLNYRPNRA----SDLPALAPP--VTPAWDDYGHS-KAFTYRIR-AR

SbAAO5 GR-QPKTA-PALAVLNYRPNRA----SKLPAVAPP--VTPAWDDYDHS-KAFTYRIR-AR

CsAAO AR-LPKTP-PGLTLLNYLPNSA----SKLPISPPP--ETPHWEDFDRS-KNFTFRIF-AA

CpAAO AR-HPNTP-PGLTLLNYLPNSV----SKLPTSPPP--QTPAWDDFDRS-KNFTYRIT-AA

CmAAO GR-HPNTP-PGLTLLNYLPNSV----SKLPTSPPP--ETPAWDDFDRS-KNFTYRIT-AA

NtAAO1 GR-EPKTP-QGLTLLNYLPNSA----SKFPTLPPP--IAPLWNDYNHS-KSFSNKIF-AL

GmAAO4 GR-PPNTP-QGLTILNYKTISA----SVFPTSPPP--ITPQWDDYNRS-KAFTYKIL-AL

GmAAO7.1 GR-KPNTP-QGLAILNYKTISA----LIFPTSPPP--ITPLWNDFEHS-KAFTKKII-AK

GmAAO6 GR-KPSTS-QGLTILNYKTISA----SIFPTSPPP--ITPLWNDFEHS-KAFTKKII-AK

GmAAO3 GR-KPNTP-QGLTILNYKPISA----SVFPTFPPP--ITPLWNDFERS-KAFTKKII-AK

GmAAO5 GRRAPNTP-QGLTILNYKPISA----SIFPISPPP--ITPIWNDFERS-KAFTKKII-AK

SbAAO6 GR-RPKTP-PALAILNYTNSKP--GRSSWPASVPP--ATPAWDNVTRS-KEFTYRIK-AR

OsAAO2 GR-HPKTV-PALAILSYGNGNAAPPPLQLPAGEPP--VTPAWNDTQRS-KAFTYSIR-AR

HvAAO3 GR-CPKTL-PATAVLRYTNSRH-----PWPGSPPP--ETPAWDDLQRS-KGFTHRIK-AR

TaAAO7 GR-PPKTL-PATAVLRYTNSRF-----PWPGSPPP--ATPAWYDLQRS-KDFARRIK-AR

TaAAO10 GR-PPKTL-PATAVLRYTNSRF-----PWPGSPPP--ATPAWYDLQRS-KGFTYRIK-AR

TaAAO12 GR-PPKTL-PATAILRYTNSRF-----PWPGSPPP--ATPAWYDLQRS-KDFTHRIN-AR

* .

NtAAO2 RPNPQGSYHYGQINITRTIKIFNSMSQVG-GKLRYGLNGISHTNGETPLKLVEY-FGATN

BnAAO RPNPQGSYHYGKINITRTIKLVNTQGKVD-GKLRFALNGVSHTEPETPLKLAEY-FGISD

OsAAO3 RPNPQGSFHYGTIATSRTLVLANSAPVLA-GQRRYAVNGVSFVVPDTPLKLVDN-YNIAN

GmAAO1 QGYIL----KPPTTSDRVIVLLNTQNNIS-EYRHWSVNNVSFTLPHTPYLISLK-ENIT-

GmAAO2 QGYIH----KPPTTSDRVIVLLNTQNNIS-EYRHWSVNNVSFTLPHTPYLIALK-ENIN-

AtAAO3 RGFIH----ALPENSDKVIVLLNTQNEVN-GYRRWSVNNVSYHHPKTPYLIALK-QNLT-

BrAAO5 KGFAH----APPENSDKVIVLLNTQNKVN-GYMRWSVNNVSYQHPTTPYLIALK-HNLT-

HvAAO2 PEHAH----PPPPRADRTILLLNSQNKID-GRVKWAINNVSFTLPHTPYLVALK-RRLR-

TaAAO5 PEHAH----PPPPRADRTILLLNSQNKID-GRIKWAINNVSFTLPHTPYLVALK-HGLL-

TaAAO1 PEHAH----PPPPRADRTILLLNSQNKID-GRIKWAINNVSFTLPHTPYLVALK-HGLL-

TaAAO3 PEHAH----PPPPRADRTILLLNSQNKID-GRIKWAINNVSFTLPHTPYLVALK-HGLL-

OsAAO4 PAHAV----PPPPTSDRTILLLNTQNKIG-GQIKWALNNVSFTLPHTPYLVAMK-RGLL-

ZmAAO2 PAYVE----PPPPRADRTILLLNTQNKID-AHTKWALNGVSFTLPHTPYLVAMK-RGLL-

SbAAO3 PAHVE----PPPPRADRTILLLNTQNKID-GHIKWALNGVSFRLPHTPYLVAMK-NGLL-

ZmAAO1 PAHVE----PPPPRADRTILLLNTQNKID-AHIKWALNGVSFTLPHTPYLVAMKRPGLL-

SbAAO2 PAHVQ----PPPPRADRTILLLNTQNKID-GHIKWALNNVSFTLPHTPYLVAMK-NGLL-

SbAAO1 PAHVQ----PPPPRADRTILLLNTQNKID-GRIKWALNNVSFTLPHTPYLVAMK-SGLL-

ZmAAO3 PDHVV----PVPPRPDRALLLLNTQNRIG-GHIRWAINGVSLAFPATPYLVSIK-RGLR-

SbAAO4 RDHVQ----PVPPRPDRALLLLNTQNRIG-GHTKWAINGVSLAFPATPYLVSMK-RGLR-

OsAAO5 PGHVE----PPPARPDRVLLLLNTQSKID-NHTKWAINGVSLSFPATPYLVAMK-HGLR-

HvAAO4 PRFVE----PMPAGADRTLLLLNTQNRID-GHIKWTINGVSLIFPATPYLVAMK-RGMT-

TaAAO6.1 PHFVE----PMPARADRTLLFLNTQNRID-GHIKWTINGVSLMFPATPYLVAMK-RGMK-

TaAAO2 PRYVV----PMPARADRTLLLLNTQNRID-GHIKWTINGVSLMFPATPYLVAMK-RGMK-

TaAAO4.1 PRFVE----PMPARADRTLLLLNTQNRID-GHIKWTINGVSLMFPATPYLVAMK-RGMK-

AtAAO2.1 MGSPS----P-PKKYRKRLILLNTQNLID-GYTKWAINNVSLVTPATPYLGSVK-YNLK-

BrAAO3 MGSPP----P-PRKFKKRLILLNTQNMID-GATKWALNNVSLVVPATPYLGSVK-YKLR-

BrAAO4 MGSPS----P-PETFDERLILLNTQNLIE-GFTKWAINNVSLAVPGTPYLGSVK-YNLR-

AtAAO1 KGYPK----P-PEKSHDQLILLNTQNLYE-DYTKWSINNVSLSVPVTPYLGSIR-YGLK-

BrAAO6 KGYPP----P-PEKSDEQLYLLNTQNLID-GHTKWAINNVSLSVTATPYIGAIR-YGLN-

BrAAO1 KGYPP----P-PEKSDEQLFLLNTQNLMD-KYTKWAINNVSLSVPATPYIGTIR-YGLK-

BrAAO2 KGYPP----P-PEKSNAQLFLLNTQNLME-GYTKWAINNLSLSVPATPYIGSIR-YGLP-

TaAAO8.1 AGTPP----P-PATSDRRIELLNTQNKLD-GHIKWSINNVSLVLPATPYLGSLK-LGLK-

HvAAO1 AGTPP----P-PATSDRRIELLNTQNKLD-GHIKWSINNVSMVLPATPYLGSLK-LGLK-

TaAAO9 AGTPP----P-PATSDRRIELLNTQNKLD-GHIKWSINNVSMVLPATPYLGSLK-LGLK-

TaAAO11 AGTPP----P-PATSDRRIELLNTQNKLD-GHIKWSINNVSLVLPATPYLGSLK-LGLK-

OsAAO1 AGVTP----PPPATSDRRIELLNTQNRMGGGHVKWSINNVSMVLPATPYLGSLK-MGLR-

ZmAAO4 AGTPP----P-PPTAARRIELLNTQNRMD-GRIRWSINNVSMVLPATPYLGSLK-MGLK-

SbAAO5 AGTAP----P-PATADRRIELLNTQNKMD-GHTKWSINNVSMVLPATPYLGSLK-MGLK-

CsAAO MGSPK----P-PVRYNRRLFLLNTQNRIN-GFMKWAINNVSLALPPTPYLAAMK-MRLN-

CpAAO MGSPK----P-PVKFNRRIFLLNTQNVIN-GYVKWAINDVSLALPPTPYLGAMK-YNLL-

CmAAO MGSPK----P-PVKSNRRIFLLNTQNVIN-GYVKWAINDVSLALPPTPYLGAMK-FNLL-

NtAAO1 MGSPK----P-PPQNHRRIILLNTQNKID-GYTKWAINNVSLVLPTQLYLGSIR-YGIN-

GmAAO4 KGTEQ----P-PQHYDRRLFLLNTQNLVD-GYTKWAINNVSLALPTTPYLGSIR-FNVN-

GmAAO7.1 MGTPQ----P-PEHSDRTQYSSST------------------------------------

GmAAO6 MGTPQ----P-PKLYDRRVFLLNTQNRVD-GFTKWSINNVSLTLPPTPYLGSIK-FKIN-

GmAAO3 MGTPQ----P-PKRSDRTIFLLNTQNRVD-GFTKWAINNVSLTLPPTPYLGSIK-FKIK-

GmAAO5 MGTPQ----P-PKRSDRTIFLLNTQNLLD-GFTKWAINNVSLTLPPTPYLGSIK-FKIN-

SbAAO6 DGTP----TPAAAAVDRRITMLNTQDWVQ-GHVKWAINHVTLSLPATPYLGAYF-YGIED

OsAAO2 KDTNRPP----PAAADRQIVLLNTQNLMD-GRYRWSINNVSLTLPATPYLGAFH-HGLQD

HvAAO3 RNAAEAPRPPPTEQVNRTIVMLNTQTLVD-GHVKWAVNNVSLTLPATPYLGAYF-YGVQG

TaAAO7 RNAAEAPPPPRTEQVSRRIVMLNTQTLVD-GHIKWAVNNVSLTLPTTPYLGAYF-YGVQG

TaAAO10 RNAAEAPPPPRTEQVNRTIVMLNTQTLVG-GHMKWAVNNVSLTLPATPYLGAYF-YGVQG

TaAAO12 RNAAEAPPPPRTEQVNRTIVMLNTQTLVG-GHMKWAVNNVSLTLPATPYLGAYF-YGVQG

.:

NtAAO2 KAFKYDLMA----------------DEA-PADPSKLTIATNVKNATYRNFVEIIFENHE-

BnAAO KVFKYDTIT----------------DDPTPEQIKNIKIEPNVLNITHRTFVEVVFENHE-

OsAAO3 V-IGWDSVP----------------ARPDGAAP---RSGTPVVRLNLHEFIEVVFQNTE-

GmAAO1 GAFDP-TPPPDGYDF-ANYDIFSVASNAN------ATSSSGIYRLKFNTTVDIILQNANT

GmAAO2 GAFDS-TPPPDGYDF-ANYDIFSVASNAN------ATSSSGIYRLKFNTTVDIILQNANT

AtAAO3 NAFDWRFTAPENYDS-RNYDIFAKPLNAN------ATTSDGIYRLRFNSTVDVILQNANT

BrAAO5 NAFDWRFTPPERYDS-KSYDIFAVPSNAN------ATMSDGIYRLKFNSTVDVVLQNANT

HvAAO2 GAFD-ERPPPETYNH-TGYDVYGVQANPN------ATTSDGLYRLAFGSVVDVVLQNANM

TaAAO5 GAFD-QCPPPETYNH-TGYDVYGVQANPN------ATTSDGLYRLAFGSVVDVVLQNANM

TaAAO1 GAFD-QRPPPETYNH-TGYDVYGVQANPN------ATTSDGLYRLAFGSVVDVVLQNANM

TaAAO3 GDFD-QRPPPETYNH-TGYDVYGVQANPN------ATTSDGLYRLAFGSVVDVVLQNANM

OsAAO4 GAFD-QRPPPETYAGAAAFDVYAVQGNPN------ATTSDAPYRLRFGSVVDVVLQNANM

ZmAAO2 DTFD-QRPPPETYAY-QGYDVYAPPQNPN------ATTSDGLYRLRFGSVVDVVLQNANM

SbAAO3 GAFD-QRPPPENYTH-QGYDVYAPPPNPN------ATISDGLYRLQFGSVVDVVLQNANM

ZmAAO1 DTFD-QRPPPETYAH-RGYDVYAVPPNPN------ATTSDGLYRLRFGSVVDVVLQNANM

SbAAO2 GAFD-QRPPPETYAH-QGYDIYAVQRNPN------ATVSDGLYRLQFGSVVDVVLQNANM

SbAAO1 GAFD-QRPPPETYAH-QGYDVYAPPPNPA------ATVSDGLYRLQFGSVVDVVLQNANM

ZmAAO3 GAYDDQRPPPDTYDY-RSYDIASP-PTAN------GTVASKVYRLALGSVVDVVLQNTVA

SbAAO4 GAYDDARPPPETYDY-RSYDIGRP-PAAN------GTVASAVYRLALGSVVDVVLQNTVA

OsAAO5 GEFD-QRPPPDSYDH-GSLNLSSP-P-AS------LAVRHAAYRLALGSVVDVVLQNTAI

HvAAO4 TAYD-QRPPSDTYDH-MSYDISAP-APTN------GTVRSPVYRLALGSVVDVVLQNSNM

TaAAO6.1 DAYE-QRPPPDMYDH-MSHDISAP-APTN------GTVGSPVYRLALGSVVDVVLQNSNM

TaAAO2 DAYE-QRPPPDMYDH-MSHEISAP-APTN------GTVGSPVYRLALGSVVDVVLQNSNA

TaAAO4.1 DAYE-QRPPPDMYDH-MSHDISAP-APTN------GTVGSPVYRLALGSVVDVVLQNSNM

AtAAO2.1 LGFNRKSPPRS-YR--MDYDIMNPPPFPN------TTTGNGIYVFPFNVTVDVIIQNANV

BrAAO3 RGFDRKSPPTT-FP--MDYDIMNPPRNRN------TTKGNGIYVFPFNVTVDVILQNANG

BrAAO4 TGFNRSSPPKD-YP--VDYDIMTPPRNRN------AKQGNVSCVFPFNVTVDVILQNANG

AtAAO1 SAYDLKSPAKKLIM--DNYDIMKPPPNPN------TTKGSGIYNFAFGIVVDVILQNANV

BrAAO6 TLSYLKSPAKE-LV--KSYDITKPPVNPT------TTKSSGIYKFPMGIVVDVILQNANV

BrAAO1 TLNYQKPPGKK-IV--EDYDITKPPVNPN------TTKGSGIYNFQLGRVVDVILQNSNV

BrAAO2 -LEYLKFPGPE-II--ENYDINHPPVNPN------TTVSSGIYNLTMGMVVDVILQNSNV

TaAAO8.1 TALA-AARPADTFG--RAYDVTRPPHNPN------TTTGDNVYVLRHNTTVDVVLQNANA

HvAAO1 TALA-AARPADTFG--RAYDVTRPPHNPN------TTTGDNVYVLRHNTTVDVVLQNANA

TaAAO9 TALT-AARPADTFG--RAYDVTRPPHNPN------TTTGDNVYVLRYNTTVDVVLQNANA

TaAAO11 TALA-AARPADTFG--RAYDVTRPPHNPN------TTTGDNVYVLRHNTTVDVVLQNANA

OsAAO1 SALPSAARPSDTFG--RGYDVMRPPANPN------TTVGDNVYVLAHNATVDVVLQNANA

ZmAAO4 STLA-AARPAETFS--REYDVTLPPPNPN------TTAGDNVYVLAHNTTVDVLLQNANA

SbAAO5 STLA-AARPAETFS--RGYDVKLPPPNPN------TTVGDNVYVLAHNTTVDVVLQNANA

CsAAO TAFN-QNPPPETFP--LNYDINNPPPNPE------TTTGNGVYKFNMGETVDVILQNANM

CpAAO HAFD-QNPPPEVFP--EDYDIDTPPTNEK------TRIGNGVYQFKIGEVVDVILQNANM

CmAAO HAFD-QNPPPEVFP--EDYDIDTPPTNEK------TKIGNGVYQFKIGEIVDVILQNANM

NtAAO1 -AFD-TKPPPDNFP--KDYDVLKQAPNSN------STYGNGVYMLKFNTTIDIILQNANA

GmAAO4 GAFD-PKSPPDNFS--MDYDILKPPLNPN------AKIGSGVYMFQFNQVVDVILQNANV

GmAAO7.1 ---------PKIEL--MDYHIFNPPVNPN------ATIGNGVYMFNLNEVVDVILQNANQ

GmAAO6 NAFD-QTPPPMNFP--QDYDIFNPPVNPN------ATIGNGVYMFNLNEVVDVILQNSNQ

GmAAO3 NAFD-KTPPPVTFP--QDYDIFNPPVNPN------ASIGNGVYMFNLNEVVDVILQNANQ

GmAAO5 NAFD-KTPPPVTFP--QDYDIFNPPVNPN------TTIGNGVYMFNLNEVVDVILQNANQ

SbAAO6 IAFDSSGESPDGYD--RRYDIKKPPGAQAPAARVPTTASDRVFRIAHGAVVDVVLQNANA

OsAAO2 SAFDASGEPPAAFP--EDYDVMRPPANNA------TTASDRVFRLRHGGVVDVVLQNANM

HvAAO3 SAFDASGEAPDGFP--DGYDIDLPPANNS----YKTRLSDRVYELPHGAVVDVVLQNADM

TaAAO7 SAFDASGEAPNGFP--GGYDIDLPPENNS----YEATLSDRVYELAHGAVVDVVLQNADM

TaAAO10 SAFDASGEAPNGFP--GGYDIDLPPANNS----YEATLSDRVYELPHGAVVDVVLQNADM

TaAAO12 SAFDASGEAPNGFP--GGYDIDLPPANNS----YETTLSDRVYELPHGAVVDVVLQNADM

::::::*

NtAAO2 ------KTIRTYHLDGYSFFAVAVEPG--RWSP--EKRKNYN----LVDGLSRNNIQVYP

BnAAO ------KSVQSWHLDGYSFFSVAVEPG--TWTP--EKRKNYN----LLDAVSRHTVQVYP

OsAAO3 ------NELQSWHLDGYDFWVVGYGNG--QWTE--NQRTTYN----LVDAQARHTVQVYP

GmAAO1 MN-KNNSETHPWHLHGHDFWVLGYGKG--KFDV-NNDTKKYN----LENPIMKNTVPVHP

GmAAO2 MT-KTNSETHPWHLHGHDFWVLGYGKG--KFDV-NNDTKKYN----LENPIMKNTVPVHP

AtAAO3 MN-ANNSETHPWHLHGHDFWVLGYGEG--KFNE-SEDPKRYN----RVDPIKKNTVAVQP

BrAAO5 MS-VNNSETHPWHLHGHDFWVLGYGEG--KFNE-MEDPKRYN----LVDPIMKNTVAVQP

HvAAO2 LA-PNNSETHPWHLHGHDFWTLGYGVG--RFDP-AAHPPAFN----LRDPVMKNTVAVHP

TaAAO5 LA-PNNSETHPWHLHGHDFWTLGYGVG--RFDP-AVHPATYN----LRDPVMKNTVAVHP

TaAAO1 LA-PNNSETHPWHLHGHDFWTLGFGVG--RFDP-AVHPATYN----LRDPVMKNTVAVHP

TaAAO3 LA-PNNSETHPWHLHGHDFWTLGYGVG--RFDP-AVHPATYN----LRDPVMKNTVAVHP

OsAAO4 LA-ANSSETHPWHLHGHDFWVLGHGAG--RFDP-AVHPAAYN----LRDPIMKNTVAVHP

ZmAAO2 LA-PNKSETHPWHLHGHDFWVLGYGIG--RFDP-AVHPASYN----LKDPVLKNTVAVHP

SbAAO3 LA-ANKCETHPWHLHGHDFWVLGYGIG--RFDP-AVHPASYN----LQDPILKNTVAVHP

ZmAAO1 LA-PNKSETHPWHLHGHDFWVLGYGIG--RFDP-AVHPASYN----LRDPILKNTVAVHP

SbAAO2 LA-ANKSETHPWHLHGHDFWVLGYGIG--RFDP-AVHPAAYN----LKDPILKNTVAVHP

SbAAO1 LA-ANKSETHPWHLHGHDFWVLGYGIG--RFDP-AVHPASYN----LKDPILKNTVAVHP

ZmAAO3 --LNNKSETHPWHLHGHDFWVLAYGDDGKKFDP-ERDTNKFN----LRDPVMKNTVALHP

SbAAO4 --LNNKSETHPWHLHGHDFWVLAYGDG--KFDP-ETDTARFN----LRDPVMKNTVALHP

OsAAO5 PPPNGRSETHPWHLHGHDFWVLGYGEG--KFVP-EVDGPGLNAASARGGAVMKNTVALHP

HvAAO4 --LNNKSETHPWHLHGHDFWVLGYGEG--KFNP-AADAWRLLN---VRDPIMKNTVPLHN

TaAAO6.1 --LNNKSETHPWHLHGHDFWVLGYGEG--KFNP-AADAWRLLN---VRDPIMKNTVPLHP

TaAAO2 --LNNKTETHPWHLHGHDFWVLGHGEG--KFNP-AADAWRLLN---VRDPIMKNTVPLHP

TaAAO4.1 --LNNKSETHPWHLHGHDFWVLGHGEG--KFNP-AADAWRLLN---VRDPIMKNTVPLHP

AtAAO2.1 LK-GIVSEIHPWHLHGHDFWVLGYGDG--KFKP-GIDEKTYN----LKNPPLRNTAILYP

BrAAO3 LD-ANASEIHPWHLHGHDFWVLGYGEG--KFRP-GIDEKTYN----LKNPPLRNTVALYP

BrAAO4 LN-ANASEIHPWHLHGHDFWVLGYGEG--KFKP-GVDEKTYN----LKNPPLRNTVALYP

AtAAO1 LK-GVISEIHPWHIHGHDFWVLGYGEG--KFKP-GIDEKTFN----LKNPPLRNTVVLYP

BrAAO6 LK-GKISEIHPWHLHGHDFWVLGYGDG--KFRP-GVDEKKYN----LKNPPLRNTVALYP

BrAAO1 LN-GRGSEIHPWHLHGHDFWVLGYGEG--KFQP-GVDDKRYN----LTNARIRNTVALYP

BrAAO2 LK-GEISEVHPWHLHGHDFWVLGYGEG--KFQP-GVDDKRYN----LTNAPLRNTVALYP

TaAAO8.1 LQ-HNVSEVHPWHLHGHDFWVLGYGEG--AYRGDAADAARLN----LANPPLRNTAVIFP

HvAAO1 LQ-HNVSEVHPWHLHGHDFWVLGYGEG--AYRGDAADAARLN----LVNPPLRNTAVIFP

TaAAO9 LQ-HNVSEVHPWHLHGHDFWVLGYGEG--AYRGDAADAARLN----LVNPPLRNTAVIFP

TaAAO11 LQ-HNVSEVHPWHLHGHDFWVLGYGEG--AYRGDAADAARLN----LVNPPLRNTAVIFP

OsAAO1 LA-RNVSEVHPWHLHGHDFWVLGYGDG--AFRGDAGDAAALN----LRNPPLRNTAVIFP

ZmAAO4 LS-RNVSEVHPWHLHGHDFWVLGYGDG--AYRGDAADEARLN----LRDPPLRNTAVIFP

SbAAO5 LS-RNVSEVHPWHLHGHDFWVLGYGDG--AYRGDAADEARLN----LRDPPLRNTAVIFP

CsAAO LN-PNMSEIHPWHLHGHDFWVLGYGEG--KFYA-PEDEKKLN----LKNPPLRNTVVIFP

CpAAO MK-ENLSETHPWHLHGHDFWVLGYGDG--KFSA--EEESSLN----LKNPPLRNTVVIFP

CmAAO MK-ENLSEIHPWHLHGHDFWVLGYGDG--KFTA--EEESSLN----LKNPPLRNTVVIFP

NtAAO1 LA-KDVSEIHPWHLHGHDFWVLGYGEG--KFSE--KDVKKFN----LKNPPLRNTAVIFP

GmAAO4 MK-GKNSEIHPWHLHGHDFWILGYGDG--KFKQ--GDDSKFN----LKNPPLRNTAVIFP

GmAAO7.1 LI-GNGSEIHPWHLHGHDFWVLGYGEG--KFKS--GDVKKFN----FTQAPLRNTAVIFP

GmAAO6 LS-VNGSEIHPWHLHGHDFWVLGYGEG--KFKL--GDEKKFN----LTHAPLRNTAVIFP

GmAAO3 LS-GSGSEIHPWHLHGHDFWILGYGEG--KFKS--GDEKKFN----LTHAPLRNTAVIFP

GmAAO5 LS-GSGSEIHPWHLHGHDFWVLGYGEG--KFKP--SDEKKFN----LTHAPLRNTAVIFP

SbAAO6 LE-EDVSESHPWHLHGHDFWVLGYGDG--VYDH-ARDSRKLD----TATPPLRNTVVLFP

OsAAO2 LR-EEVSETHPWHLHGHDFWVLGYGDG--RYDP-AAHAAGLN----AADPPLRNTAVVFP

HvAAO3 LR-HNESETHPWHLHGHDFWVLGYGEG--RYR-----SERLN----TEDPPLRNTVVVFP

TaAAO7 RR-DNDSETHPWHLHGHDFWVLGYGEG--RYT----GGERLN----TEDPPLRNTVVVFP

TaAAO10 RR-DNDSETHPWHLHGHDFWVLGYGEG--RYGG-GGGGERLN----TEDPPLRNTVVLFP

TaAAO12 RR-DNDSETHPWHLHGHDFWVLGYGEG--RYTS---GRERLN----TEDPPLRNTVVVFP

: :*:.*:.*: :. . : ::. :

NtAAO2 NSWAAIMLTFDNAGMWNLRSEMWEKTYLGEQLYFSVLSPSRSLRDEYNIPDNHPLCGIVK

BnAAO KCWAAILLTFDNCGMWNVRSENTERRYLGQQLYASVLSPEKSLRDEYNMPETSLQCGLVK

OsAAO3 NGWSAILVSLDNQGMWNLRSANWDRQYLGQQLYMRVWTPQQSFSNEYSIPTNAILCGRAA

GmAAO1 FGWTALRFRTDNPGVWAFHCHIESHFYMGMGVVFEE--GIERV-GK--LPSSIMGCGQTR

GmAAO2 FGWTALRFRTDNPGVWAFHCHIESHFYMGMGVVFEE--GVERV-GK--LPSSIMGCGQTR

AtAAO3 FGWTALRFRADNPGVWSFHCHIESHFFMGMGIVFES--GIDKV-SS--LPSSIMGCGQTK

BrAAO5 YGWTALRFRADNPGVWAFHCHIESHFFMGMRIVFAS--GIDRV-AN--LPSSIMGCGQTK

HvAAO2 YGWTALRFRADNPGVWAFHCHIEAHFFMGMGVAFEE--GIERV-GK--LPEEITRCVSKK

TaAAO5 FGWTALRFRADNPGVWAFHCHIEAHFFMGMGVAFEE--GIERV-GD--LPEEIRRCVSTK

TaAAO1 FGWTALRFRADNPGVWAFHCHIEAHFFMGMGVAFEE--GIERV-GD--LPEEIRRCVSTK

TaAAO3 FGWTALRFRADNPGVWAFHCHIEAHFFMGMGVAFEE--GIERV-GD--LPEEIRRCVSTK

OsAAO4 FGWTALRFRADNPGVWAFHCHIEAHFFMGMGIVFEE--GVERV-GE--LPPEIMGCGKTR

ZmAAO2 YGWTALRFKADNPGVWAFHCHIEAHFFMGMGIVFEE--GIQRV-AS--LPPEIMGCGETN

SbAAO3 YGWTAVRFKADNPGVWAFHCHIEAHFFMGMGIVFEE--GIQRV-AN--LPPEIMGCGKTK

ZmAAO1 YGWTALRFRADNPGVWAFHCHIESHFFMGMGIAFEE--GVDRV-AP--LPPQIMGCGKTR

SbAAO2 YGWTALRFKADNPGVWAFHCHIESHFFMGMGIVFEE--GVERV-AQ--LPKEITGCGMTK

SbAAO1 YGWTALRFKADNPGVWAFHCHIESHFFMGMGIVFEE--GVERV-AQ--LPQEIMGCGKTK

ZmAAO3 RGWTAVRFVADNPGVWLFHCHIEAHVYMGMGLVFEE--GVDKV-GR--LPKSIMGCGRSR

SbAAO4 KGWTAVRFVADNPGVWLFHCHIEAHVYMGMGVVFEE--GVDKV-GR--LPKSIMGCGRSR

OsAAO5 MGWTAVRFRASNPGVWLFHCHLEAHVYMGMGVVFEE--GVDVL-PR--LPASIMGCGRTK

HvAAO4 DGWTAVRFRADNPGVWLFHCHIEAHVFMGMGVVFEE--GIERV-GK--LPPSIMGCGQSK

TaAAO6.1 DGWTAVRFRADNPGVWLFHCHVEAHVFMGMGVVFEE--GVERV-GR--LPSSIMGCGRSK

TaAAO2 DGWTAVRFRADNPGVWLFHCHVEAHVFMGMGVVFEE--GVKRV-GR--LPSSIMGCGRSK

TaAAO4.1 DGWTAVRFRADNPGVWLFHCHVEAHVFMGMGVVFEE--GVERV-GR--LPSSIMGCGRSK

AtAAO2.1 YGWTAIRFVTDNPGVWFFHCHIEPHLHMGMGVVFAE--GLNRI-GK--VPDEALGCGLTK

BrAAO3 YGWTALRFVTDNPGVWFFHCHIEPHLHMGMGVVFAE--GLNRI-GK--VPDEALGCGLTK

BrAAO4 YGWTALRFVTDNPGVWFFHCHIEPHLHMGMGVVFAE--GLNRI-GK--VPDEALGCGLTK

AtAAO1 FGWTAIRFVTDNPGVWFFHCHIEPHLHMGMGVVFVE--GVDRI-GKMEIPDEALGCGLTR

BrAAO6 FGWTALRFVTDNPGVWFFHCHIEPHLHMGMGVVFAE--GVDRI-AKMDIPDEVLGCGLTR

BrAAO1 YGWTALRFVTDNPGVWFFHCHIEPHLHMGMGVVFAE--GVDQI-AKMNIPNEVLGCGLTR

BrAAO2 YGWTALRFVTDNPGVWFFHCHIEPHLHMGMGVVFAE--GVDQI-AKMNIPREALGCGSTR

TaAAO8.1 YGWTALRFVADNPGVWAFHCHIEPHLHMGMGVIFAE--AIDRV-GK--VPKEAVSCGATA

HvAAO1 YGWTALRFVADNPGVWAFHCHIEPHLHMGMGVIFAE--AIERV-GK--VPKEAVSCGATA

TaAAO9 YGWTALRFVADNPGVWAFHCHIEPHLHMGMGVIFAE--AIDRV-GK--VPKEAVSCGATA

TaAAO11 YGWTALRFVADNPGVWAFHCHIEPHLHMGMGVIFAE--AIDRV-GK--VPKEAVSCGATA

OsAAO1 YGWTAIRFVADNPGVWAFHCHIEPHLHMGMGVIFAE--AVDRV-SE--LPKAAVSCGATA

ZmAAO4 YGWTMLRFVADNPGVWAFHCHIEPHLHMGMGVIFAE--AVDLV-AK--VPNEAVSCGATA

SbAAO5 YGWTMLRFVADNPGVWAFHCHIEPHLHMGMGVIFAE--AVDLV-GK--VPNEAVSCGATA

CsAAO YGWTAIRFVADNPGVWAFHCHIEPHLHMGMGVVFAE--GVHMV-GM--IPPKALACGSTA

CpAAO YGWTAIRFVADNPGVWAFHCHIEPHLHMGMGVVFAE--GVEKV-GR--IPTKALACGGTA

CmAAO YGWTAIRFVADNPGVWAFHCHIEPHLHMGMGVVFAE--GVEKV-GR--IPTKALACGGTA

NtAAO1 FGWTALRFVTDNPGVWAFHCHIEPHLHMGMGVIFAE--GVHLV-KK--IPKEALACGLTG

GmAAO4 HGWTALRFKADNPGVWAFHCHIEPHLHMGMGVIFAE--AVQNVTST--IPRDAFACGILK

GmAAO7.1 YGWTALRFKADNPGVWAFHCHIEPHLHMGMGVVFAE--GVHKV-GK--IPREALTCGLTG

GmAAO6 YGWTALRFKADNPGVWAFHCHIEPHLHMGMGVIFAE--GVHKV-GK--IPREALTCGLTG

GmAAO3 YGWTALRFKADNPGVWAFHCHIEPHLHMGMGVIFAE--AVQKV-GK--IPRDALTCGLTG

GmAAO5 YGWTALRFKADNPGVWAFHCHIEPHLHMGMGVIFAE--GVHKV-GK--IPRDALTCGLTG

SbAAO6 HGWTVLRFVADNPGVWAFHCHIEPHLHLGMGVIFAE--GMEKL-RELNVPREAITCGEAK

OsAAO2 HGWTALRFVANNTGAWAFHCHIEPHLHMGMGVVFVE--GEDRM-HELDVPKDAMACGLVA

HvAAO3 HGWTAIRFVADNVGAWAFHCHIEPHLHMGMGAVFVE--GVDKM-RELDVPREAMMCGVIK

TaAAO7 HGWTAIRFVADNVGAWAFHCHIEPHLHMGMGAVFVE--GAHMI-RELDVPREAMMCGVIR

TaAAO10 HGWTAIRFVADNVGAWAFHCHIEPHLHMGMGAVFVE--GAHMI-RELDVPRETMMCGVIR

TaAAO12 HGWTAIRFVADNVGAWAFHCHIEPHLHMGMGAVFVE--GAHKI-RELDVPREAMMCGVIR

*: : . .* * * .:. : .:* . :* *

NtAAO2 GLSMPAPYKA-------------

BnAAO NTPKPVNPYAGA-----------

OsAAO3 GLGH-------------------

GmAAO1 GFHRP------------------

GmAAO2 GFHGP------------------

AtAAO3 R----------------------

BrAAO5 RLV--------------------

HvAAO2 GGQH-------------------

TaAAO5 GGHH-------------------

TaAAO1 GGGH-------------------

TaAAO3 GGGH-------------------

OsAAO4 GGH--------------------

ZmAAO2 GGHR-------------------

SbAAO3 GGH--------------------

ZmAAO1 GGH--------------------

SbAAO2 GGH--------------------

SbAAO1 GGH--------------------

ZmAAO3 T----------------------

SbAAO4 TLP--------------------

OsAAO5 GHHY-------------------

HvAAO4 GLH--------------------

TaAAO6.1 GLH--------------------

TaAAO2 GLH--------------------

TaAAO4.1 GLH--------------------

AtAAO2.1 QFLMNRNRN--------------

BrAAO3 QFLMNRNNP--------------

BrAAO4 QFLMNRNNP--------------

AtAAO1 KWLMNRGRP--------------

BrAAO6 KWLMNQGRH--------------

BrAAO1 EFLMNRGRH--------------

BrAAO2 EMFMNQGRH--------------

TaAAO8.1 TALMNGDHL--------------

HvAAO1 TALMNGDHP--------------

TaAAO9 TALMNGDHL--------------

TaAAO11 TALMNGDHL--------------

OsAAO1 TALMAGAGGHV------------

ZmAAO4 TALMAGGHL--------------

SbAAO5 TALMAGGHV--------------

CsAAO LV-KNYPRLP-------------

CpAAO KSLINNPKNP-------------

CmAAO KSLINNP----------------

NtAAO1 KMLMSNKHN--------------

GmAAO4 KFLNKEHN---------------

GmAAO7.1 KKLIENGRY--------------

GmAAO6 KKLVENGHY--------------

GmAAO3 K-MLGNRHY--------------

GmAAO5 NKLVGNRHY--------------

SbAAO6 TASLSLAPAVAPSRP--------

OsAAO2 RTAATPLTPATPLPPSPAPAP--

HvAAO3 TSA-AVLTPSKPRSPAPAPAPAP

TaAAO7 TTV-AALTPAKPGSPAPAP----

TaAAO10 TAA-ASLTPAKPGSPAPSAHG--

TaAAO12 TTA-AALTPAKPGSSAPAPSAHR
