# Supplementary material for: Transcript Profiling Reveals the Presence of Abiotic Stress and Developmental Stage Specific Ascorbate Oxidase Genes in Plants
Source: Front Plant Sci. 2017 Feb 17;8:198. doi: 10.3389/fpls.2017.00198 (PMC5314155; doi:10.3389/fpls.2017.00198)
Supplement: Supplementary file 3 [file Data_Sheet_2.docx]

**Transcript profiling reveals the presence of abiotic stress and developmental stage specific ascorbate oxidase genes in plants**

**Rituraj Batth, Kapil Singh, Sumita Kumari ^*^, Ananda Mustafiz^*^**

^*^Correspondence: Ananda Mustafiz: [amustafiz@sau.ac.in](mailto:amustafiz@sau.ac.in)

^*^Correspondence: Sumita Kumari: [sumitaslsjnu@gmail.com](mailto:sumitaslsjnu@gmail.com)

**Figure S2:** Multiple sequence alignment of *OsAAO1-OsAAO5* genes of rice at nucleotide level using Clustal Omega (1.2.4). The positions of forward and reverse primers used in qRT-PCR are also marked on nucleotide sequence of respective *AAO* genes. Forward and reverse primer binding site are highlighted in yellow and blue color, respectively.

OSAAO1 AT------------GAGGCTGTCGTCCCTGCTGTTCTTGGTGTGCTTCTTCACTGTCGCC 48

OSAAO2 ATGGCGGCCGCCGTGCAGCTGCTCGTCGTCGCCGCCGCCGCCGCCATGGCGGCGGCGTGC 60

OSAAO3 ATGGCGCC--TCCTCCGGCTGCAGC-TGCGGCGTTGGCGGCGTGCATTCTCGCCGTCGCC 57

OSAAO4 AT------------GATGCGGTGTAGCGACCGCCTCCTCTGCAGCCTCTTCCTCGCCGCC 48

OSAAO5 AT------------GAGGACGTGGCGGCTCGCCGTGCTGGCGTGCC---TGTGCGCCGCC 45

** * * * * *

OSAAO1 ATGTCGCAATGCGCGGCGGCGGCGAAGGCGAGGCACTTCAGGTGGGAGGTGAGCAACAT- 107

OSAAO2 TGCGCCGGCATGGCGGCGGCGGCGGCGACGGTGGAGGTGACGTGGGACGTGGAGTACGT- 119

OSAAO3 GCCACCCTCGCCGGCGCCGACGACCCCTACCGCTTCTTCACCTGGAACGTCACCTACGGC 117

OSAAO4 GCGCTGTTCGGCGTCGCCGCCGCGGCGACGCGGCGCCACGACTGGGACATCAGCTACCA- 107

OSAAO5 GCGGCGGCGGCGCCGGCGGAGGCGAAGACGCACCACCACACGTGGAACATCACGTACCA- 104

** * * *** * * **

OSAAO1 --GTTCTGGTCGCCGGACTGCGAGGAGAAGGTGGTGATCGGCATCAATGGCCAGTTCCCC 165

OSAAO2 --ACTGTGGGCGCCGGACTGCCAGCAGCGGGTGATGATCGGGATAAACGGCAGGTTCCCG 177

OSAAO3 AGCATCAACCCTCTCGGCTCCACCCCGCAGCAGGGCATCTTGATCAATGGCCAGTTTCCT 177

OSAAO4 --GTTCACGTCGCCCGACTGCGTACGCAAGCTGGCCGTCACCATCAACGGCCACACGCCG 165

OSAAO5 --GTACAAGTCGCCGGACTGCTTCAGGAAGCTCGCCGTGACCATCAACGGCGAGTCCCCC 162

* * * ** * * * ** ** *** **

OSAAO1 GGGCCGACCATCCGCGCCAAGGCCGGCGACACCATCGTCGTCCACCTCAAGAACGGCCTG 225

OSAAO2 GGGCCCAACATCACCGCGCGCGCCGGCGACGTGATCAGCGTCACCATGAACAACAAGATG 237

OSAAO3 GGGCCGCGAATCGACTGCGTGACGAACGACAACATCATCGTGAACGTGTTCAACAACCTG 237

OSAAO4 GGCCCCACCATCCGCGCCGTCCAGGGGGACACCATCGTCGTCAACGTCAAGAACTCGCTG 225

OSAAO5 GGGCCGACCATCCGCGCCGCCCAGGGCGACACCCTCGTCGTCACCGTCCACAACATGCTC 222

** ** *** * *** ** *** * * *** *

OSAAO1 CACACCGAGGGCGTCGTCATCCACTGGCACGGCATCAGACAGATCGGGACACCATGGGCG 285

OSAAO2 CACACCGAGGGCGTCGTCATCCACTGGCACGGCATCAGACAGTTTGGCACGCCGTGGGCG 297

OSAAO3 GACGAGCCGT---TCCTCCTGACATGGAATGGCATCAAGCAGCGCAAGAACTCGTGGCAG 294

OSAAO4 CTGACGGAGAACGTCGCCATCCACTGGCACGGCATCCGGCAGATCGGCACGCCGTGGGCG 285

OSAAO5 GACACCGAGAACACCGCCATCCACTGGCACGGCATCCGCCAGATTGGCAGCCCGTGGGCT 282

* * * * *** * ****** *** * * ***

OSAAO1 GATGGCACGGCATCGATCTCCCAGTGCGCCATCAACCCTGAAGAAACCTTCACCTATCGC 345

OSAAO2 GACGGGACGGCATCGATATCCCAGTGCGCAGTGAACCCGGGCGAGACGTTCGTCTACAAG 357

OSAAO3 GACG---GCGTGCTGGGCACCAACTGCCCAATCCCCCCTGGCGCCAACTACACCTACAAG 351

OSAAO4 GACGGCACGGAGGGCGTCACCCAGTGCCCCATCCTCCCCGGCGACACCTTCGCCTACACC 345

OSAAO5 GACGGCACCGCCGGCGTCACGCAGTGCCCCATCCTCCCCGGCGAGACCTTCACCTACAGA 342

** * * * * *** * * *** * * * * * ***

OSAAO1 TTCGTTGTCGACAAGCC---GGGGACGTACTTCTACCACGGCCACTACGGGATGCAGAGG 402

OSAAO2 TTCGTCGCCGACAAGCC---GGGCACCTACTTCTACCACGGCCACTTCGGGATGCAGCGC 414

OSAAO3 TTCCAGGCCAAGGACCAGATCGGCACCTTCGTCTACTTCCCCTCCGTCGCCATGCACCGC 411

OSAAO4 TTCGTCGTCGACCGCCC---GGGCACCTACATGTACCACGCCCACTACGGGATGCAGCGC 402

OSAAO5 TTCGTCGTTGACAGGCC---TGGAACGTACATGTACCACGCGCACTACGGCATGCAGCGC 399

*** * * * ** ** * * * *** * * ** ***** *

OSAAO1 GCGGCGGGGCTGTACGGGTCGCTGATCGTGGACGTCGCCGACGG---------------C 447

OSAAO2 GCCGCGGGCCTGTACGGTTCCCTCATCGTCCTCGACTCGCCGGAGCAGCCCGAGCCGTTC 474

OSAAO3 GCCGCCGGCGGCTTCGGCGCGCTCAACGTCTACCAGCGCCCGGCCATCCCCGT------- 464

OSAAO4 TCGGCGGGCCTCAACGGCATGATCGTCGTCGAGGTCGCCCCCGGCGCCGCCGGCGACGGC 462

OSAAO5 GTGGCGGGGCTGGACGGCATGCTCGTCGTGTCGGTGCCCGACGG---------------C 444

** ** *** * *** *

OSAAO1 GACGAGGAGCCGTTCAAGTACGACGGCGAGATCAACCTGCTGCTCAGCGACTGGTACCAC 507

OSAAO2 CGCCACCAGTACGACGACGGCGGCGAGCTCCCCATGATGCTCCTCAGCGACTGGTGGCAC 534

OSAAO3 -----CCCGTACCCGCCGCCCGCCGGCGACTTCACGCTCCTCGTCGGCGACTGGTACAAG 519

OSAAO4 GAGCGGGAGCCGTTCAGGTACGACGGGGAGCACACCGTGCTGCTCAACGACTGGTGGCAC 522

OSAAO5 GTCGCCGAGCCCTTCGCCTACGACGGCGAGCACACCGTCCTCCTCATGGACTGGTGGCAC 504

* ** ** ** * ** ** ******* *

OSAAO1 GAGAGCATCTACACCCAGATGGTCGGCCTCTCCTCCA------ACCCCTTCCGATGGATC 561

OSAAO2 CAGAACGTCTACGCCCAGGCCGCCGGACTCGACGGCAAGGACAGGCACTTCGAGTGGATC 594

OSAAO3 GCCGGGCATAAGCAGCTGAGGCAGGCGCTCGACGCCG------GCGGCGGTGGCGCGCTC 573

OSAAO4 CGGAGCACGTACGAGCAGGCGGCGGGGCTCGCGTCCG------TGCCCATGGTGTGGGTC 576

OSAAO5 CAGAGCGTGTACGAGCAAGCCGTCGGCCTCGCCTCCG------TCCCCATGGTGTTCGTC 558

* * * *** * * **

OSAAO1 GGCGAGCCGCAGTCATTGCTGATCAATGGGAGGGGCCAGTTCAACTGCTCGCTGGCGGCG 621

OSAAO2 GGCGAGCCCCAGACGATCTTGATCAATGGGAGAGGACAGTTCGAGTGCACGCTGGGGCCA 654

OSAAO3 CCGCCGCCCGACGCGCTGCTCATC------------------------------------ 597

OSAAO4 GGCGAGCCGCAGTCGCTGCTCATCAACGGGCGCGGCCGGTTCGTGAACTGCTCGTCCTCG 636

OSAAO5 GGCGAGCCCCAGTCGCTTCTGATCAACGGCAGAGGCGTGTTCAACTGCTCGCCGCCGGCG 618

*** * * * * ***

OSAAO1 GCGCACACGCCGGGCG-------------------------------------------- 637

OSAAO2 GCGAGGAAGAGCTTTGAGAAGCTCCTCAACGAGAACGTGGAGACCTGCGTCGACGACCAG 714

OSAAO3 ------------------------------------------------------------ 597

OSAAO4 CCGGCGACGG-------------------------------------------------- 646

OSAAO5 GCCAGCAATGGCGGTG-------------------------------------------- 634

OSAAO1 -------------------------------------CCAAGCAGTGCGCCGCCGCCGGC 660

OSAAO2 AAGATGTGCAGCGACCAGGAGAAGTGCCTGAGGAGGAGCGAGTGCGGGCCGTACTGCCCC 774

OSAAO3 ------------------------------------------------------------ 597

OSAAO4 -------------------------------------CGGCGTCGTGCAACGTG---TCG 666

OSAAO5 -------------------------------------GCGCGGCGTGCAATGCGTTTGGC 657

OSAAO1 AACCGGCACTGCGCTCCGGTGATCCTCCCCGTCCTTCCCAACAAAACGTACAGGCTCAGG 720

OSAAO2 AGGAGCCAGTGCGCCCCTGTCGTGTTCAATGTCGAGCAGGGGAAGACTTACCGCCTTAGG 834

OSAAO3 AATGGCATGCCGTCGGCGGCGGCGTTCGTCGGCGACCAGGGGAGGACGTACCTGTTCAGG 657

OSAAO4 CACCCGGACTGCGCGCCGGCGGTGTTCGCCGTGGTGCCCGGGAAGACGTACCGCTTCCGC 726

OSAAO5 GGCGAGTGCGGGTGGCCGACGCTGTTCACCGCCTCGCCGGGGAAGACGTACCGCCTCCGC 717

* ** * * * ** *** * *

OSAAO1 GTCGCGAGCACCACCTCGCTCGCTTCCCTCAACCTCGCCGTCGGGAATCACAAGCTGACG 780

OSAAO2 ATCGCCAGCACCACCTCCCTTTCTCTCCTCAACGTCAAGATTCAAGGGCACAAGATGACG 894

OSAAO3 GTGTCCAATGTCGGGGTGAAGACGTCCGTCAATGTCAGGATCCAGGGGCACTCGCTGAGG 717

OSAAO4 GTCGCCAGCGTCACCTCCCTCTCCGCGCTCAACTTCGAGATCGAGGGGCACGAGATGACG 786

OSAAO5 ATCGGCAGCCTGACGTCGCTGGCGTCGCTGAGCTTCGAGATCGAGGGGCACACGATGACG 777

* * * * * ** * *** * *** *

OSAAO1 GTGGTGGAGGCCGACGGGAACTACGTGGAGCCGTTCGCCGTCGACGACATCGACATCTAC 840

OSAAO2 GTGGTGGAGGCCGACGGGAACCACGTGGAGCCGTTCGTGGTCGACGACATCGACATCTAC 954

OSAAO3 TTGGTGGAGGTGGAGGGGACGCACCCGGTGCAGAACGTGTACGACTCGCTCGACGTCCAT 777

OSAAO4 GTGGTGGAGGCCGACGGTCACTACGTGAAGCCGTTCGTGGTGAAGAACCTCAACATCTAC 846

OSAAO5 GTGGTGGAGGCCGACGGGTACTACGTCACGCCGGTGGTGGTCAAGAACCTCTTCATCTAC 837

********* ** ** ** ** * * * ** * ** *

OSAAO1 TCCGGCGACAGCTACTCCGTGCTGCTGACGACGGACCAGGACACGTCGGCGAACTACTGG 900

OSAAO2 TCCGGCGAGAGCTACTCCGTCCTCCTCAAGGCCGACCAGAAGCCGGCGAGCTACTGGATC 1014

OSAAO3 GTCGGCCAGTCGGTGGCGTTCCTCGTCACGCTCGACAAGGCGGCGCAGGACTACGCCGTC 837

OSAAO4 TCCGGCGAGACCTACTCCGTCCTCATCACCGCCGACCAGGACCCCAACCGCAACTACTGG 906

OSAAO5 TCCGGCGAGACCTACTCCGTGCTCGTCACCGCCGACCAGGACCCGTCCCGGAGCTACTGG 897

**** * * * ** * * *** ** * *

OSAAO1 GTCAGCGTCGGCGTGCGCGGCCGGCAGCCCAGGACGGCGCCAGCGCTGGCCGTGCTCAAC 960

OSAAO2 T---CCGTCGGCGTCAGGGGGCGCCACCCCAAGACGGTGCCGGCGCTCGCCATCCTCAGC 1071

OSAAO3 GTGGCGTCCGCGCGGTTCAGCCCGGGCGCGTCGCCGCTGATGGCGACGGGGACGCTGCAC 897

OSAAO4 CTCGCCTCCAACGTCGTCAGCCGCAAGCCGGCCACCCCCACCGGCACCGCCGTCCTCGCC 966

OSAAO5 GCGGCGTCGCACGTCGTCAGCCGCGACCCCACCAAGACGGCGCCGGGCAGGGCCGTCGTC 957

* * * * *

OSAAO1 TACCGCCCCAAC------------CGCGCGTCCAGGCTGCCGGCGGCGGCGCCGCCGGCC 1008

OSAAO2 TACGGCAACGGCAACGCGGCGCCGCCGCCGCTCCAGCTGCCCGCCGGCGAGCCCCCCGTG 1131

OSAAO3 TACAGCAGCGCC------GTGTCCAGGGCGCCCGGCCCGCTCCCGGCGCCGCCGCCGGAG 951

OSAAO4 TACTACGGCGGCCGCCGCAA---CAGCCCCCGCGCCCGCCCGCCCACGCCGCCGCCCGCC 1023

OSAAO5 AGGTACGCCTCC------------GCCGCCGTGGATCACCCGCGCACGCCGCCGCCGACC 1005

* * * * * * *** **

OSAAO1 ACCCCGGCGTGGGACGACTTCGCGCGCAGCAAGGCGTTCACGTACCGCATCCTCGGCCGC 1068

OSAAO2 ACGCCGGCGTGGAACGACACACAGCGCAGCAAGGCCTTCACCTACAGCATCAGGGCGCGC 1191

OSAAO3 CAGGCGGAGTGGTCGATGAACCA---GGCGAGGTCGTTCCGGTGGAACCTGACGGCGAGC 1008

OSAAO4 GGCCCGGCGTGGAACGACACCGCCTACCGCGTCCGCCAGAGCCTCGCCACCGTCGCGCAC 1083

OSAAO5 GGGCCACGGTGGAACGACACGGCGAGCAGGGTGGCGCAGAGCAGGTCGTTCGCCGCGCTG 1065

* **** *

OSAAO1 GCCGGCGTCACGCCGCCGCCGCCGGCGACGTCGGACCGGCGC---------------ATC 1113

OSAAO2 AAGGACACCAACCGGCCGCCGCCGGCGGCCGCCGACCGGCAG---------------ATC 1236

OSAAO3 GCGGCGAGGCCCAACCCGCAGGGGTCGTTCCACTACGGCACCATCGCGACGTCGAGGACG 1068

OSAAO4 CCGGCGCACGCCGTGCCCCCGCCGCCGACCTCCGACCGCACC---------------ATC 1128

OSAAO5 CCGGGGCACGTCGAGCCGCCGCCGGCGAGGCCCGACCGCGTT---------------CTC 1110

* ** * * * ** ** *

OSAAO1 GAGCTGCTCAACACGCAGAACCGGATGGGCGGCGGGCACGTGAAGTGGTCGATCAACAAC 1173

OSAAO2 GTCCTGCTCAACACGCAGAACCT---CATGGACGGGCGCTACAGGTGGTCCATCAACAAC 1293

OSAAO3 CTGGTGCTCGCCAACTCCGCGCCGGTGCTCGCCGGGCAGCGCCGGTACGCCGTCAACGGC 1128

OSAAO4 CTGCTGCTCAACACGCAGAACAA---GATCGGCGGGCAGATCAAGTGGGCGCTCAACAAC 1185

OSAAO5 CTCCTCCTCAACACGCAGAGCAA---GATCGACAACCACACCAAGTGGGCCATCAACGGC 1167

* *** ** * * * ** * ***** *

OSAAO1 GTGTCCATGGTGCTCCCGGCGACGCCGTACCTGGGGTCCCTCAAGATGGGGCTGAGGTCG 1233

OSAAO2 GTGTCCCTGACGCTGCCGGCGACGCCGTACCTGGGCGCCTTCCACCACGGCCTCCAGGAC 1353

OSAAO3 GTGTCGTTCGTCGTCCCCGACACGCCGCTCAAGCTCGTGGACAACTACAACATC------ 1182

OSAAO4 GTCTCCTTCACGCTGCCTCACACGCCGTACCTCGTCGCCATGAAGCGCGGGCTCCTCGGC 1245

OSAAO5 GTCTCCCTCAGCTTCCCGGCGACGCCGTACCTCGTCGCCATGAAGCACGGCCTCCGCGGC 1227

** ** * * ** ****** * * *

OSAAO1 GCGCTCCCGTCGGCGGCGAGGCCGT---CCGACACGTTCGGGCGCGGGTACGACGTGATG 1290

OSAAO2 AGCGCGTTCGACGCGTCCGGCGAGCCGCCGGCGGCGTTCCCGGAGGACTACGACGTGATG 1413

OSAAO3 ---------------------------------------GCCAATGTCATCGGCTGGGAC 1203

OSAAO4 GCCTTCGACCAGCGCCCGCCGCCGGAGACGTACGCCGGCGCCGCCGCGTTCGACGTGTAC 1305

OSAAO5 GAGTTCGACCAGCGGCCGCCGCCGGACAGCTACGACCACGGGAGCCTCAACCTCTCCTC- 1286

* *

OSAAO1 CGGCCGCCGGCGAACCCGAACACC---------ACGGTGGGCGACAACGTGTACGTGCTC 1341

OSAAO2 AGGCCGCCGGCGAACAACGCGACG---------ACGGCGAGCGACAGGGTGTTCCGGCTG 1464

OSAAO3 AGCGTCCCGGCGAGGCCCGACGGCGCGGCGCCGCGGTCGGGGACGCCGGTGGTGAGGCTC 1263

OSAAO4 GCCGTGCAGGGGAACCCCAACGCC---------ACCACCAGCGACGCGCCGTACCGGCTC 1356

OSAAO5 --CCCGCCGGCG------AGCCTC---------GCCGTGCGCCACGCCGCGTACCGCCTC 1329

* ** * * * **

OSAAO1 GCGCACAACGCGACGGTGGACGTGGTGCTCCAGAACGCGAACGC---GCTGGCGCGGAAC 1398

OSAAO2 CGACACGGCGGCGTGGTGGACGTGGTGCTCCAGAACGCCAACAT---GCTGAGGGAGGAG 1521

OSAAO3 AACCTGCACGAGTTCATCGAGGTGGTG---------------------TTCCAGAACACG 1302

OSAAO4 CGGTTCGGCTCCGTCGTCGACGTCGTGCTCCAGAACGCCAACATG---CTGGCGGCGAAC 1413

OSAAO5 GCCCTGGGCTCGGTGGTCGACGTGGTGCTGCAGAACACGGCGATCCCGCCGCCGAACGGG 1389

* * ** ** *** *

OSAAO1 GTCAGCGAGGTGCACCCGTGGCACCTCCACGGGCACGACTTCTGGGTGCTGGGCTACGGC 1458

OSAAO2 GTGAGCGAGACGCACCCGTGGCACCTCCACGGCCACGACTTCTGGGTGCTCGGCTACGGC 1581

OSAAO3 GAGAACGAGCTGCAGTCTTGGCATCTCGATGGATATGACTTCTGGGTTGTTGGGTATGGC 1362

OSAAO4 AGCAGCGAGACGCACCCGTGGCACCTCCACGGCCACGACTTCTGGGTGCTCGGCCACGGC 1473

OSAAO5 CGGAGCGAGACGCACCCGTGGCACCTCCACGGGCACGACTTCTGGGTGCTCGGCTACGGC 1449

* **** *** * ***** *** * ** * *********** * ** * ***

OSAAO1 GACGGCGCGTTCCGGGGCGACGCCGGCGACGCGG---------CGGCGCTGAACCTGAGA 1509

OSAAO2 GACGGCCGGTACGACCCGGCGGCGCACGCGGCGGG------------GCTCAACGCCGCC 1629

OSAAO3 AATGGTCAGTGGACTGAGAATCAGCGGACAA---------------CCTACAACTTGGTT 1407

OSAAO4 GCCGGCCGGTTCGACCCGGCGGTGCACCCGGCGGC------------GTACAACCTCAGG 1521

OSAAO5 GAGGGCAAGTTCGTGCCGGAGGTGGACGGGCCGGGTCTGAACGCGGCGAGCGCGAGGGGC 1509

** **

OSAAO1 AACCCGCCGCTGCGGAACACGGCGGTGATCTTCCCGTACGGGTGGACGGCGATCCGGTTC 1569

OSAAO2 GACCCGCCGCTGCGGAACACGGCGGTGGTCTTCCCGCACGGGTGGACGGCGCTTCGGTTC 1689

OSAAO3 GATGCGCAAGCGAGGCATACAGTTCAGGTTTACCCGAATGGATGGTCGGCAATCTTGGTG 1467

OSAAO4 GACCCAATCATGAAGAACACGGTGGCGGTGCACCCGTTCGGGTGGACGGCGCTCCGGTTC 1581

OSAAO5 GGCGCCGTGATGAAGAACACGGTGGCGCTGCACCCGATGGGGTGGACGGCGGTGAGGTTC 1569

* * * * ** * * * **** ** *** **** * * *

OSAAO1 GTGGCGGACAACCCCGGGGTGTGGGCGTTCCACTGCCACATCGAGCCGCACCTCCACATG 1629

OSAAO2 GTCGCCAACAACACCGGCGCGTGGGCGTTCCACTGCCACATCGAGCCGCACCTCCACATG 1749

OSAAO3 TCATTGGACAACCAGGGGATGTGGAACCTGAGGTCGGCGAACTGGGACCGGCAATACCTC 1527

OSAAO4 AGGGCGGACAACCCCGGCGTGTGGGCGTTCCACTGCCACATCGAGGCGCACTTCTTCATG 1641

OSAAO5 AGGGCGAGCAACCCGGGCGTGTGGCTGTTCCACTGCCACCTGGAGGCCCACGTGTACATG 1629

**** ** **** * * * * * *

OSAAO1 GGCATGGGCGTCATCTTCGCCGAGGCCGTCGACCGCGTCAGCGAGCTCCCCAAGGCGGCC 1689

OSAAO2 GGCATGGGCGTCGTCTTCGTCGAGGGGGAGGACAGGATGCACGAGCTCGACGTGCCCAAG 1809

OSAAO3 GGCCAGCAGCTGTAC----ATGAGAGTGTGGACGCCGCAGCAGAGCTTCTCCAATGAGT- 1582

OSAAO4 GGCATGGGCATCGTCTTCGAGGAGGGCGTCGAGCGCGTCGGCGAGCTGCCGCCGGAGATC 1701

OSAAO5 GGCATGGGCGTGGTGTTCGAGGAGGGCGTCGACGTGCTGCCGCGGCTGCCGGCGTCCATC 1689

*** * * *** * ** ***

OSAAO1 GTCTCCTGCGGCGCCACCGCCACCGCGCTCATGGCCGGCGC--------CGGCGGCCACG 1741

OSAAO2 GACGCCATGGCGTGCGGCCTCGTCGCCAGGACGGCCGCCACGCCGCTCACCCCGGCAACG 1869

OSAAO3 ----ACAGTATCCCGACCAACGCCATACTCTG--CGGTAG---------AGCTGCC---- 1623

OSAAO4 ATGGGCTGCGGCAAAACCAGGGGCGGCCACTGA--------------------------- 1734

OSAAO5 ATGGGGTGCGGCCGCACCAAGGGCCATCACTACTAA------------------------ 1725

* *

OSAAO1 TGTGA------------------------------------------------------- 1746

OSAAO2 CCGCTGCCTCCGTCGCCGGCGCCGGCGCCATGA--------------------------- 1902

OSAAO3 ----GGCCTTGGACAC--------------TGA--------------------------- 1638

OSAAO4 ------------------------------------------------------------ 1734

OSAAO5 ---------------------------------AAACTTGTGTTATCCAAAAAAAAAAAAC 1753

OSAAO1 ------------------------------------------------------------ 1746

OSAAO2 ------------------------------------------------------------ 1902

OSAAO3 ------------------------------------------------------------ 1638

OSAAO4 ------------------------------------------------------------ 1734

OSAAO5 TACTAAAAACTTGTTGTTCTTATACTTCATGGTGACGAACGTCCAACAGCTTAGTCATCA 1813

OSAAO1 ------------------------------------------------------------ 1746

OSAAO2 ------------------------------------------------------------ 1902

OSAAO3 ------------------------------------------------------------ 1638

OSAAO4 ------------------------------------------------------------ 1734

OSAAO5 TGTGTCAAAACATTACTATAAGGGTTGGGATTTGGGAAGTTTGTTTTCATTAATTTTGTA 1873

OSAAO1 ----------------------------------------------- 1746

OSAAO2 ----------------------------------------------- 1902

OSAAO3 ----------------------------------------------- 1638

OSAAO4 ----------------------------------------------- 1734

OSAAO5 AACATAGTGACCACATTGGTCAATTGTTTTAATATTGAATAAGAGCA 1920
